# The Effect of Diet and Dietary Supplements on Iron Status of Active Females: A Systematic Review and Meta-analysis of Interventional Trials

**DOI:** 10.1007/s40279-026-02464-x

**Published:** 2026-06-10

**Authors:** Emma McCarthy, Josh P. Pekler, Tina Tran, Lauren E. Deluca, Trina Fresco, Jarrad Hampton-Marcell, Beatriz Penalver Bernabe, Karrie Hamstra-Wright, Mary Dawn Koenig, Danielle Colegrove, Lisa Tussing-Humphreys

**Affiliations:** 1 Department of Kinesiology and Nutrition, University of Illinois Chicago, 1919 West Taylor Street, Chicago, IL 60612 USA; 2 College of Osteopathic Medicine, Kansas City University, Joplin, MO 64804 USA; 3 Department of Biological Sciences, University of Illinois Chicago, Chicago, IL 60612 USA; 4 Department of Biomedical Engineering, University of Illinois Chicago, Chicago, IL 60612 USA; 5 Department of Human Development Nursing Science, University of Illinois Chicago, Chicago, IL 60612 USA; 6 Department of Athletic Medicine, University of Illinois Chicago, Chicago, IL 60612 USA

## Abstract

**Background:**

Iron deficiency is reported in about 15–35% of all female athletes. In addition to oral iron supplements, food-based approaches and dietary supplements have gained interest in preventing and treating iron deficiency in the active female population.

**Objectives:**

This systematic review and meta-analysis aimed to evaluate the effect of diet and dietary supplement interventions on iron status, performance, and inflammatory markers in active females of reproductive age.

**Data Source:**

The search was conducted using PubMed, Scopus, CINAHL, Embase, and Rehabilitation & Sports Medicine Source up to March 2026.

**Data Extraction:**

Interventional trials that examined the impact of diet or dietary supplements on at least one biomarker of iron status in active females of reproductive age were included.

**Data Analysis:**

All meta-analyses were conducted in R version 2025.05.0 + 496 using the metafor package. Separate random-effects meta-analyses were performed for hemoglobin and ferritin outcomes.

**Results:**

Oral iron supplementation in the form of ferrous sulfate resulted in a significant increase in hemoglobin (mean difference [MD] = 0.42 g/dL; 95% confidence interval [CI]: 0.10 to 0.74; *p* = 0.0109; *I*^2^ = 56.6%) and ferritin (MD = 12.61 ng/mL; 95% CI: 8.29 to 16.92; *p* < 0.0001; *I*^2^ = 57.38%).

**Conclusion:**

Meta-analysis revealed that ferrous sulfate supplementation significantly improved hemoglobin and ferritin levels in active females.

**Systematic Review Registration:**

PROSPERO registration no. CRD42024554679.

:

**Supplementary Information:**

The online version contains supplementary material available at https://doi.org/10.1007/s40279-026-02464-x.

## Key Points

**Table:** 

This review provides a comprehensive summary of diet and dietary supplement strategies to improve iron status among active females, including newer dietary interventions such as those exploring the role of the gut microbiome in iron absorption.	
Our meta-analysis revealed that ferrous sulfate supplementation increased ferritin concentration by an average of 12.61 ng/mL.	
This change is clinically relevant and may move athletes with low baseline iron status across iron-deficiency stages.	

## Introduction

Iron is one of many minerals essential for optimum athletic performance. Of its several roles in the human body, the most notable for physical performance is its ability to facilitate oxygen transport and energy production [1]. Iron deficiency impairs energy metabolism, and oxygen cannot efficiently reach tissues during activity. This may manifest as shortness of breath and muscle fatigue [2, 3]. If left untreated, iron deficiency can lead to iron deficiency anemia with side effects beyond decreased performance, such as an increased risk of infections [1]. Increased energy expenditure, reduced energy intake, exercise-induced hemolysis, exercise-induced inflammation, and iron loss in sweat contribute to the risk of negative iron balance in heavily trained individuals [4]. While iron’s importance and role are well documented in sports nutrition, it remains a common nutrient of concern, especially among female athletes. Iron deficiency is reported in about 15–35% of all female athletes [4]. Active females are at a higher risk of iron deficiency owing to iron loss through menstruation [4].

While necessary for adaptation, the inflammatory response to exercise can impair systemic iron metabolism [5]. During physical activity, muscle glycogen is used for energy. As glycogen stores deplete, interleukin (IL)-6 is released into the circulation from muscle and adipose tissue [6]. The degree of IL-6 release is influenced by training status, exercise duration, and carbohydrate intake [7, 8]. The rise in IL-6 post exercise stimulates the release of hepcidin [5]. Hepcidin, an iron regulatory hormone produced in and released by the liver, decreases the amount of iron transported into the circulation from diet and body stores by binding to the iron exporter, ferroportin-1. Ferroportin is located on the surface of hepatocytes, enterocytes, and other iron-handling tissues. Therefore, inflammation can impair access to iron from the diet and body stores. Compared with baseline measurements, hepcidin levels increase up to 2.5-fold, peaking 3–6 h post exercise in athletes [9]. This mechanism raises the question of proper timing of iron intake to optimize absorption from the diet.

Attention to adequate nutrition and recovery is essential to support the demanding physical and psychological workload of physical training and sport.

With the previously mentioned variables impacting the iron metabolism of each athlete uniquely, it is difficult to establish a one-size-fits-all intervention. Thus, several iron-enhancing techniques have been explored. The most invasive and expensive approach is intravenous iron infusions and intramuscular iron injections. These interventions are typically reserved for severe cases of iron deficiency anemia as they can bypass the gut and rapidly replete body iron stores [10]. They are prescribed and administered with the guidance of a physician; however, “no needle” policies in professional sports organizations may contraindicate their use [10]. Others have explored less invasive strategies such as standard oral iron supplements, iron-rich foods, iron-fortified foods, and nutrition counseling with varied success [11, 12]. Traditional iron supplements may cause gastrointestinal discomfort and potentially disrupt the gut microbiome [13]. In the context of systemic inflammation, hepcidin is elevated, which may reduce iron bioavailability from both dietary sources and body iron stores [14]. The absorption rate from whole-food sources will also range depending on whether the food source contains heme or nonheme iron. Other compounds in food, such as vitamin C, can improve iron absorption, while polyphenols found in tea, for example, can hinder iron absorption [15]. While iron is available in the food matrix or alone as an oral supplement, there are barriers to the success of these interventions.

This systematic review aims to evaluate dietary approaches to maintaining and improving the iron status of premenopausal active females. While a recent meta-analysis by Šmid et al. [11] explored the effect of oral iron supplements in healthy adult athletes, our study goes beyond this review and is far more comprehensive. We summarize the existing evidence base and explore recent initiatives that include efforts targeting the gut microbiome to optimize iron digestion and absorption while limiting the unwanted side effects of oral iron supplements. We also explore the outcomes of the intervention on athletic performance and systemic inflammation.

## Methods

This systematic review followed Preferred Reporting Items for Systematic Reviews and Meta Analyses (PRISMA) guidelines [16] and was registered with PROSPERO (CRD #42024554679).

### Literature Search

A database search strategy was developed with a University of Illinois Chicago research librarian. PubMed, Scopus, CINAHL, Embase, and Rehabilitation & Sports Medicine Source were the databases chosen for the literature search. Relevant search terms fell into one of three concepts: iron, female athlete, and diet intervention; see the supplementary material for the complete list of search terms for each database.

### Selection Criteria

Inclusion criteria were defined as females of reproductive age (12–52 years) [17] who are physically active, defined as “tier 2” or above, training at least three times per week and identifying with a specific sport or activity [18], a diet or dietary supplement intervention, single-arm, parallel arm, crossover, randomized or nonrandomized interventional trials, and included iron-related outcomes such as hemoglobin (Hb), hematocrit (HCT), ferritin, or serum iron (SI). Exclusion criteria were defined as: studies involving only males or with results not stratified by sex, females aged less than 12 years or greater than 52 years, pregnant/lactating, sedentary, and minimally active populations defined as 300 min or less of moderate-intensity activity a week [18], observational study design, those with animal or cell models, reviews, or literature such as conference abstracts and proceedings. The team used the Covidence software program [19] for systematic reviews with all four screening stages: title and abstract, full text, data extraction, and quality assessment. With each stage, a publication review required two independent reviewers and a consensus meeting to clarify conflicts. Our research team comprised four reviewers (E.E.M., J.P.P., T.T., L.E.D., T.F). Upon importing the database results, the software removed 4318 duplicates, leaving 2441 publications for title and abstract screening. The team removed 2210 publications deemed irrelevant, leaving 231 articles for the full-text portion of the screening.

### Data Extraction and Quality Assessment

In total, 42 articles met the eligibility criteria and moved on to the data extraction stage. Here, two reviewers for each article collected details on study design, population characteristics, intervention type, and study outcomes of interest. The reviewers also assessed each article’s quality using the Cochrane Risk of Bias tool (RoB2) [20]. This tool uses the following five domains to determine the risk of bias: randomization, deviations from intended interventions, missing outcome data, measurement of outcome, and selection of reported results.

### Meta-Analysis Methods

Studies that met the following criteria were included in the meta-analysis: parallel-arm randomized controlled trials (RCTs); availability of arm-level pre-/and post-means, standard deviations, and sample sizes for hemoglobin and ferritin outcomes; and intervention with ferrous sulfate as the sole iron compound. Only ferrous sulfate had sufficient studies for meta-analysis. The Hb outcome was reported in eight RCTs and ferritin was reported in seven RCTs. No crossover designs were included. When data were reported as medians and ranges or interquartile ranges, approximate means and standard deviations were calculated using methods proposed by Wan et al. [21]. For studies that reported duration as a range, we used the midpoint value. Hemoglobin values reported in g/L were converted to g/dL by dividing by 10 to ensure unit consistency across studies.

A random-effects meta-analysis was performed using the rma() function from the metafor package in R [22]. Effect sizes were calculated as mean differences with corresponding sampling variances using escalc(). Between-study heterogeneity was assessed using *I*^2^, *τ*^2^, and Cochran’s *Q* test. Sensitivity analyses were conducted using the leave1out() function to evaluate the influence of individual studies on the overall effect size and heterogeneity. Meta-regression was used to explore the influence of dose, intervention duration, and baseline iron status. *p* < 0.05 was considered statistically significant.

## Results

### Literature Review

#### Data Extraction and Quality Assessment

**Fig. 1:**
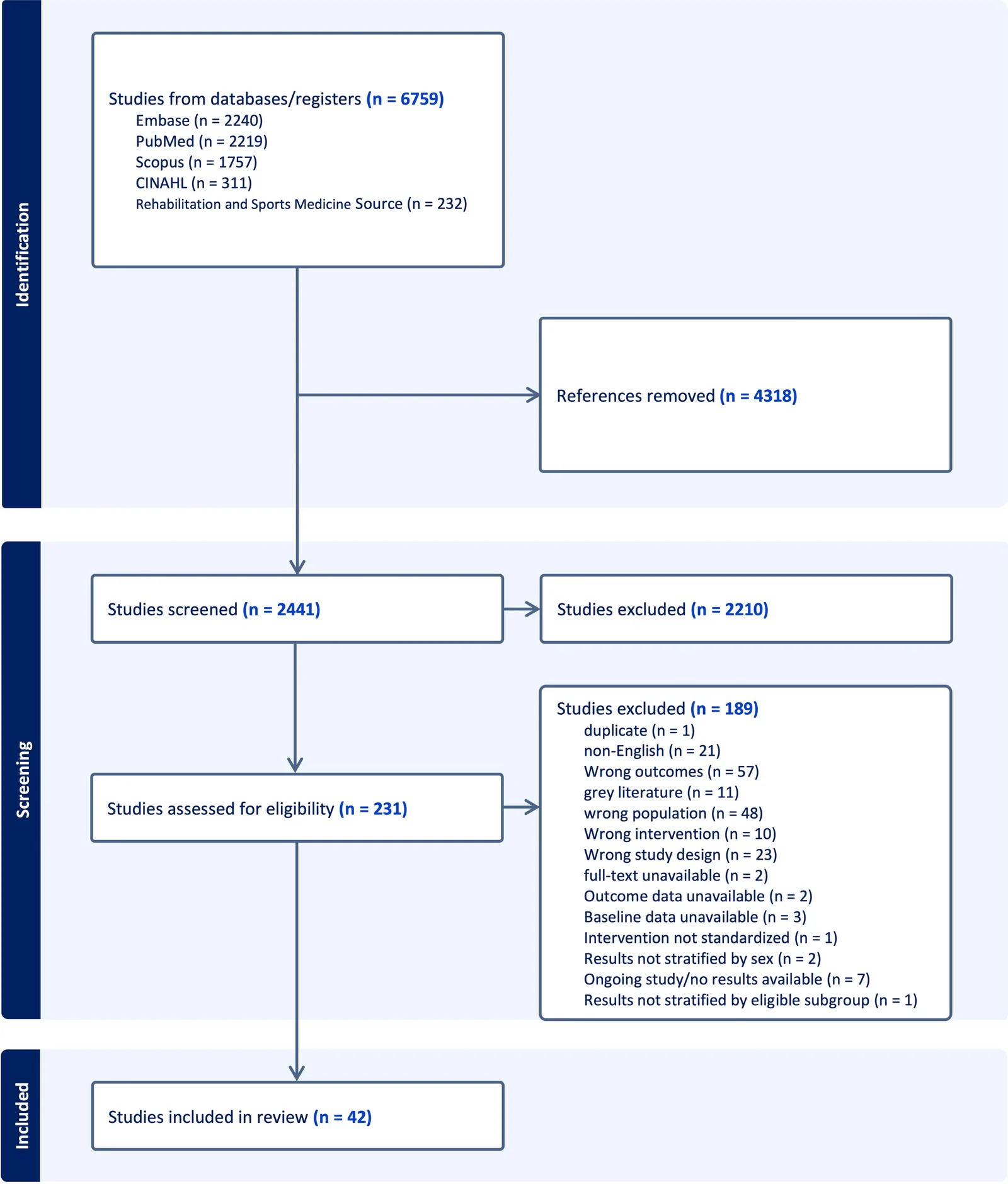
PRISMA flow diagram of the study selection process

Population, Intervention, Comparison, Outcomes, and Study (PICOS) criteria are presented in Table 1. Our search strings retrieved a total of 6759 results. In total, 42 articles met the eligibility criteria and moved on to the data extraction stage (Fig. 1). Table 2 presents the quality assessment results using the Cochrane Risk of Bias tool. Overall, risk of bias was low across the five domains. Risk of bias in domain 1 and 2 had the most concern, with 16 of 42 selected trials having some concerns or high risk of bias arising from the randomization process and 9 of 42 having some concerns or high risk owing to deviations from the intended intervention.

**Table 1:** PICOS criteria for inclusion of studies

Parameter	Criteria
Population	Active females aged 12–52 years
Intervention	Diet or dietary supplement
Comparator	Placebo versus intervention or single arm (baseline and endpoint)
Outcomes	Primary: At least 1 biomarker of iron status was measured before and after the intervention. Secondary: VO_2_max, blood lactate, hepcidin, interleukin-6, and C-reactive protein
Study design	Intervention studies

**Table 2:** Quality assessment according to Cochrane Risk of Bias 2 (ROB2) tool

Study	Randomization	Deviation	Missing data	Outcome measurement	Reporting
Cialdella-Kam et al. 2014 [41]	High	Low	Low	Low	Low
Sha et al. 2018 [43]	High	Some concerns	Low	Low	Low
Durkalec-Michalski et al. 2021 [51]	High	Low	Low	Low	Low
Mugambi 2023 [50]	Some concerns	Low	Low	Low	Low
Schoene et al.1983 [34]	Low	Low	Low	Low	Low
Matter et al. 1987 [59]	Some concerns	Some concerns	High	Low	Some concerns
Rowland et al. 1988 [33]	Low	Low	Low	Low	Low
Yoshida et al. 1990 [64]	Low	Low	Low	Low	Low
Karamizrak et al. 1996 [37]	High	Some concerns	Low	Low	Low
Łagowska 2018 [49]	Some concerns	Low	Low	Low	Low
Cieślicka et al. 2023 [42]	Low	Some concerns	Low	Low	Low
Kapoor et al. 2023 [36]	Low	Low	Low	Low	Low
Booth et al. 2014 [23]	Low	Low	Low	Low	Low
Dellavalle and Haas 2014 [25]	Low	Low	Low	Low	Low
Shaw et al. 2023 [44]	Low	Low	Low	Low	Low
Kang and Matsuo 2004 [40]	Low	Low	Low	Low	Low
Pate et al.1979 [31]	Low	Low	Low	Low	Low
Alaunyte et al. 2014 [46]	High	Some concerns	Low	Low	Low
Aguilo et al. 2021 [54]	High	High	Low	Low	Low
Plowman and McSwegin 1981 [62]	Some concerns	Some concerns	Low	Low	Low
Miller et al.1983 [39]	Low	Low	Low	Low	Low
Ishizaki et al. 2006 [53]	Some concerns	Low	Low	Low	Low
Kawano et al. 2002 [57]	Low	Some concerns	Low	Low	Low
McClung et al. 2009 [29]	Low	Low	Low	Low	Low
Karl et al. 2010 [48]	Low	Low	Low	Low	Low
Pitsis et al. 2004 [61]	Some concerns	Low	Low	Low	Low
Stroescu et al. 2001 [45]	Low	Low	Low	Low	Low
Koikawa et al. 2008 [58]	Low	Low	Low	Low	Low
Hennigar et al. 2016 [47]	Low	Low	Low	Low	Low
Schulte et al. 2024 [38]	High	Low	Low	Low	Low
Mielgo-Ayuso et al. 2015 [30]	Low	Low	Low	Low	Low
Brigham et al. 1993 [24]	Low	Low	Low	Low	Low
Graja et al. 2021 [52]	Some concerns	Low	Low	Low	Low
Flynn et al. 2003 [35]	Some concerns	Low	Low	Low	Low
SanAtanasio et al. 2023 [55]	Low	High	Low	Low	Low
LaManca 1993 [28]	Some concerns	Low	Low	Low	Low
Klingshirn et al.1992 [27]	Low	Low	Low	Low	Low
Fogelholm et al. 1992 [26]	Low	Low	Low	Low	Low
Powell and Tucker 1991 [32]	Low	Low	Low	Low	Low
Sandroni et al. 2022 [63]	Low	Low	Low	Low	Low
McCormick et al. 2020 [60]	Some concerns	Low	Low	Low	Low
Axling et al. 2020 [56]	Low	Low	Low	Low	Low

#### Study Characteristics

Organized by intervention category, Table 3 presents study characteristics and outcomes of interest with reported statistical significance. Intervention categories include oral iron supplements (OIS) (*n* = 18), other supplements (*n* = 5), food-based iron (*n* = 5), dietary lifestyle counseling (*n* = 5), and combination therapy, defined as an intervention that uses an oral iron supplement in at least one group and incorporated an additional dietary component, either administered alongside the iron or used as a comparator in another study arm (*n* = 9). Of the 42 trials in this review, 7 were conducted in Asia [36, 40, 43, 53, 57, 58, 64], 3 in Africa [50, 52, 59], 17 in North America [24, 25, 27–29, 31–34, 38, 39, 41, 44, 47, 48, 62, 63], 11 in Europe [26, 30, 37, 42, 45, 46, 49, 51, 54–56], and 4 in Australia [23, 35, 60, 61]. The sample sizes ranged from 5 to 171 participants.

**Table 3:** Summary of published diet and dietary supplement interventions targeting iron status in female athletes (*n* = 42 publications)

Study	Study design and sample size	Population characteristics (activity, age)	Study arms (description, frequency, and duration)	Iron-related outcomes	Systemic inflammatory and performance outcomes
**Oral iron supplements**					
**Iron salts**					
Booth et al. 2014 [23]	RCT: parallel-arm *n* = 49	Military Arm 1: 20.0 ± 2.0 years Arm 2: 20.0 ± 3.1 years	1: Placebo (glucose capsule) 2: 18 mg of elemental iron as ferrous gluconate 1x/day, 13 weeks	Hb: ∅^1 2 a^ Ferritin: ↓^1^, ∅^2 a^	
Brigham et al. 1993 [24]	RCT: crossover *n* = 25	Swimming 19.1 ± 1.1 years	1: Placebo 2: 39 mg as 295 mg ferrous sulfate 1x/day, 13 weeks	Hb: ↓^1^, ∅^2^, ↑^a^ Ferritin: ∅^1^,↑^2 a^	
Dellavalle and Haas 2014 [25]	RCT: parallel-arm *n* = 31	Rowing Arm 1: 19.7 ± 0.9 years Arm 2: 19.8 ± 1.1 years	1: Iron 100 mg/d ferrous sulfate 2: Placebo (lactose capsule) 2x/day, 6 weeks	Hb: ∅^1 2 a^ Ferritin: ∅^1 2 a^	
Fogelholm et al. 1992 [26]	RCT: parallel-arm *n* = 31	Mixed competitive sports Arm 1: 24 years (median) Arm 2: 21 years	1: 100 mg ferrous sulfate 2: Placebo 1 × /day, 8 weeks	Hb: ∅^1 2^, ↑^a^ Ferritin:↑^1a^, ∅^2^ HCT: ∅^1 2^, ↑^a^	VO_2_max:∅^1 2 a^ Lactate: ∅^1 a^, ↓^2^
Klingshirn et al.1992 [27]	RCT: parallel-arm *n* = 18	Running 28.83 ± 4.5 years	1: 160 mg ferrous sulfate capsule equivalent to 50 mg of elemental iron 2: Control capsule 2 × /day, 8 weeks	Hb: ∅^1 2 a^ Ferritin: ↑^1 2 a^ HCT: ∅^1 2 a^ SI: ∅^1 2 a^	VO_2_max: ∅^1 2 a^ Lactate: ∅^1 2 a^
LaManca 1993 [28]	Nonrandomized experimental study *n* = 20	Running Arm 1: 28 ± 6 years Arm 2: 28 ± 5 years	1: 159 mg ferrous sulfate (50 mg elemental iron) 2: Placebo capsule 2x/day, 8 weeks	Hb: ↑^1^, ∅^2 a^ Ferritin: ↑^1 a^, ∅^2^ HCT: ∅^1 2^, ↑^a^ SI: ∅^1 2 a^	VO_2_max: ∅^1 2^ ↑^a^ Lactate: ∅^1 2 a^
McClung et al. 2009 [29]	RCT *n* = 171	Military Arm 1: 20.8 ± 4.42 years Arm 2: 20.4 ± 4.2 years	1: Placebo cellulose capsules 2: 100 mg ferrous sulfate capsules 1x/day, 8 weeks	Hb: ∅^1 2 a^ Ferritin: ↓^1^, ∅^2^, ↑^a^	
Mielgo-Ayuso et al. 2015 [30]	RCT *n* = 22	Volleyball 27.0 ± 5.6 years	1: Control no treatment 2: 325 mg of ferrous sulfate, equivalent to 105 mg of elementary iron 1x/day, 11 weeks	Hb: ↓^1^, ∅^2^, ↑^a^ Ferritin: ↓^1^, ∅^2^, ↑^a^ HCT: ∅^1 2 a^ SI: ↓^1^, ∅^2^, ↑^a^	
Pate et al.1979 [31]	RCT *n* = 26	Mixed intercollegiate sports NR: participants recruited from intercollegiate teams	1: 167 mg ferrous sulfate (50 mg elemental iron) 2: Lactose placebo 1 × /day, 5–9 weeks	Hb: ∅^1 2 a^ HCT: ∅^1 2 a^ SI: ∅^1 2 a^	
Powell and Tucker 1991 [32]	Pre-test, post-test single-blind crossover *n* = 10	Running (cross-country) NR: participants recruited from Colorado State University cross-country team	1: 650 mg ferrous sulfate; 130 mg elemental iron capsules 2: Placebo lactose-charcoal capsule 1 × /day, 2 weeks	Hb: ∅^1 2 a^ Ferritin: ∅^1 2 a^ HCT: ∅^1 2 a^ SI: ∅^1 2 a^	VO_2_max: ∅^1 2 a^ Lactate: ∅^1 2 a^
Rowland et al. 1988 [33]	RCT *n* = 14	Running (cross-country) NR: participants of high school age	1: Capsules of ferrous sulfate, 975 mg/d 2: Placebo starch capsules 1 × /day, 4 weeks	Hb: ∅^1 2 a^ Ferritin: ↑^1 a^, ∅^2^	
Schoene et al.1983 [34]	RCT *n* = 15	Mixed individual and team sports 20 ± 15.65 years	1: Ferrous Sulfate 300 mg pill 2: Placebo pill 3x/day, 2 weeks	Hb: ↑^1^, ∅^2 a^ Ferritin: ↑^1^, ∅^2 a^	VO_2_max: ∅^1 2 a^ Lactate: ↓^1^,∅^2 a^
Flynn et al. 2003 [35]	Nonrandomized experimental study *n* = 22	Running (long-distance) and triathlon Arm 1: 25.8 ± 7.0 years Arm 2: 28.2 ± 9.0 years	1: 350 mg ferrous gluconate (105 mg elemental iron) 2: Placebo capsules 2 × /day, 8 weeks	Ferritin: ↑^1 a^, ∅^2^ SI: ∅^1 2 a^	
Kapoor et al. 2023 [36]	RCT: parallel-arm *n* = 40	Mixed endurance sports 41.8 ± 1.5 years (SEM)	1: Dietary iron sachet with 3.6 mg of ferric pyrophosphate in granule with 1. 37 g carbohydrate and 1.8 mg sodium 2: Placebo: dextrin (carbohydrate 1.42 g) 1x/day, 4 weeks	Hb: ∅^1 2 a^	
Karamizrak et al. 1996 [37]	Nonrandomized experimental study *n* = 5	Mixed team and individual sport 18.6 ± 3.8 years	1: 350 mg and 175 mg of ferrous fumarate 2 × /day, 3 weeks	Hb: ↑^1^ Ferritin: ↑^1^ SI: ↑^1^	
**Chelated iron**					
Schulte et al. 2024 [38]	Other: prospective cohort study *n* = 33	Track and Field and soccer Arm 1: 19.7 ± 1.2 years Arm 2: 20 ± 1.1 years	1: 25 mg of elemental iron as Ferrochel chelate 3 × /week, 8 weeks 2: 25 mg of elemental iron as Ferrochel chelate 1 × /day, 8 weeks	Hb: ∅^1 2^, NR^a^ Ferritin: ↑^1 2^, ∅^a^	
**Heme iron**					
Miller et al.1983 [39]	Nonrandomized experimental study *n* = 21	Running 19–45 years	1: Bovine heme–iron 600 mg 2: Placebo 1 × /day, 8 weeks	Hb: ↓^1^, ∅^2^, NR^a^ HCT: ∅^1 2^, NR^a^ SI: ∅^1 2^, NR^a^	VO_2_max: ∅^1 2 a^
**Unspecified iron form**					
Kang and Matsuo 2004 [40]	RCT: parallel-arm *n* = 25	Soccer Arm 1: 22.6 ± 2.0 years Arm 2: 23.8 ± 2.8 years	1: (40 mg elemental iron) taken in 15-ml solution as tolerated 2: Control appeared identical to the active agent and was taken in 15-ml solution 1 × /day, 4 weeks	Hb: ∅^1 a^, ↓^2^ Ferritin: ↑^1^, ∅^2 a^ HCT: ↓^1 2^ ∅ ^a^	
**Other supplements**					
Cialdella-Kam et al. 2014 [41]	Nonrandomized experimental study *n* = 8	Endurance training 22.6 ± 3.3 years	1: CHO-PRO supplement: 325 mL of Gatorade® Nutrition Drink (54 g carbohydrates, 20 g protein) 1 × /day, 6 months	Ferritin: ∅^1^ SI: ∅^1^	VO_2_max: ∅^1^
Cieślicka et al. 2023 [42]	RCT (placebo controlled clinical trial) *n* = 20	Basketball Arm 1: 17.09 ± 1.24 years Arm 2: 16.0 ± 0.67 years	1: Bovine colostrum 3.2 g, 4 capsules 2: Control 3.2 g powdered milk, 4 capsules 2 × /day, 6 months	Hb: ∅^1 2 a^ Ferritin: ∅^1 2 a^ HCT: ∅^1 2 a^	Hepcidin: ∅^1 2 a^
Sha et al. 2018 [43]	RCT *n* = 20	Soccer Arm 1: 13.7 ± 1.06 years Arm 2: 12.18 ± 0.86 years	1: Control standard water 2: 1.5–2 L hydrogen-rich water treatment Daily, 8 weeks	Hb: ∅^1^, ↑^2 a^	IL-6: ∅^1 2^ ↓^a^
Shaw et al. 2023 [44]	RCT *n* = 26	Running 34.6 ± 9.7 years	1: 130 g powder made from regular peas 2: 120 g powder made from peas bred to have low phytic acid 3: Control 125 g nonpea control (maltodextrin) 3x/day, 8 weeks	Hb: ∅^1 2 3 a^ Ferritin: ∅^1 2 3 a^	CRP: ∅^1 2 3 a^ VO_2_max: ∅^1 2 3 a^ Lactate: ∅^1 2 a^
Stroescu et al. 2001 [45]	RCT *n* = 14	Gymnastics 14.9 ± 1.3 years	1: 1 g/kg of bodyweight received soy protein powder dissolved in 250 ml water 2: Control group: placebo beverage consisting of 10 g sugar, 3 g cocoa, and 250 mL water 2 × /day, 4 months	Hb: ∅^1 2 a^	
**Food-based iron**					
Alaunyte et al. 2014 [46]	Nonrandomized experimental study *n* = 11	Running 32 ± 7 years	1: Teff bread provided 7.0 ± 3.3 mg iron a day which contributed to 45% of the daily recommendation value for dietary iron Daily, 6 weeks	Ferritin: ∅^1^	
Hennigar et al. 2016 [47]	RCT *n* = 54	Military 18–42 years	1: Placebo food bar 2: Ca/vitamin D-fortified food bar 2 × /day, 9 weeks	Hb: ↓^1^, ∅^2 a^ Ferritin: ↓^1 2^, ∅^a^	
Karl et al. 2010 [48]	RCT *n* = 124	Military Arm 1: 21 ± 4 years Arm 2: 21 ± 3 years	1: Placebo food bars containing 1.6 mg native iron 2: Food bars fortified with encapsulated 27.9 mg ferrous sulfate 2 × /day, 9 weeks	Hb: ↑^1 2 a^ Ferritin: ∅^1 2 a^	Hepcidin: ∅^1 2 a^ CRP: ↓^1^, ∅^2^, ↑^a^ IL-6: ∅^1 2 a^
Łagowska 2018 [49]	Nonrandomized experimental study *n* = 41	Mixed team and individual sports 17.8 ± 2.2 years	1: Iron-rich diet Daily, 12 weeks	Hb: ∅^1^ Ferritin: ↑^1^ HCT: ∅^1^ SI: ↑^1^	
Mugambi 2023 [50]	Nonrandomized experimental study *n* = 11	Running (marathon) 18–36 years	1: (Athle-food) was prepared with pearl millet, soybean, and milk powder Daily, 2 months	Hb: ↑^1^	
**Dietary lifestyle changes**					
Durkalec-Michalski et al. 2021 [51]	Nonrandomized experimental study *n* = 11 female participants	CrossFit 30.5 ± 3.3 years	1: Ketogenic ≥ 75% daily energy from fat, 1.7 g of protein per kilogram of body mass, and up to 5% energy from carbohydrates (10-day menu) Daily, 4 weeks	Hb: ↓^1^ HCT: ∅^1^	VO_2_max: ∅^1^ Lactate: ∅^1^
Graja et al. 2021 [52]	Nonrandomized experimental study *n* = 12	Handball 16.5 ± 0.5 years	1: Ramadan intermittent fasting Daily, 1 month	Hb: ∅^1^ HCT: ∅^1^	
Ishizaki et al. 2006 [53]	Nonrandomized experimental study *n* = 8	Rhythmic gymnastics 18.6 ± 0.5 years	1: Fixed diet: common Japanese foods containing 15 mg iron and 1500 kcal energy Daily, 4 weeks	Hb: ∅^1^ Ferritin: ↑^1^ HCT: ↓^1^	
Aguilo et al. 2021 [54]	Other: quasi-experimental intervention design *n* = 24	Gymnastics 14.1 ± 2.3 years	1: Performance nutrition counseling for gymnasts and/or their parents (45 or 90 min per session) 1 × /month, 8 months	Hb:∅^1^ Ferritin:↑^1^ HCT:∅^1^ SI:∅^1^	
SanAtanasio et al. 2023 [55]	RCT *n* = 19	Soccer Arm 1: 19 ± 1.4 years Arm 2: 22.5 ± 4.8 years	1: Controlled diet 2: Exchange diet Daily, 12 weeks	Hb: ↑^1^ ∅^2 a^ Ferritin: ∅^1 2 a^ HCT: ∅^1 2 a^ SI: ∅^1 2 a^	
**Combination therapy**					
Axling et al. 2020 [56]	RCT *n* = 39	Mixed competitive sports Arm 1: 22.3 ± 3.5 years Arm 2: 21.6 ± 6 years	1: *Lactobacillus plantarum* 299v; 1010 CFU + 20 mg ferrous fumarate 2: 20 mg ferrous fumarate 1 × /day, 12 weeks	Hb: ∅^1 2 a^ Ferritin: ↑^1 2^, ∅^a^ SI: ∅^1 2 a^	Hepcidin: ∅^1 2 a^ CRP: ∅^1 2 a^ VO_2_max: ∅^1 2 a^ Lactate: ∅^1 2 a^
Kawano et al. 2002 [57]	RCT *n* = 13	Rhythmic gymnastics 19.3 ± 1.4 years	1: 250 mL of milk with breakfast and dinner + 4 mg iron pyrophosphate 2: 250 mL of milk between breakfast and dinner + 4 mg iron pyrophosphate 2 × /day, 3 months	Hb: ∅^1 2^, ↑^a^ Ferritin: ↑^1 a^,∅^2^ HCT: ∅^1 2^, ↑^a^ SI: ∅^1 2 a^	
Koikawa et al. 2008 [58]	RCT *n* = 16	Running Arm 1: 20.3 ± 1.3 years Arm 2: 20.1 ± 1.5 years	1: Lactoferrin tablets containing 0.45 g of bovine lactoferrin + 5 mg ferric pyrophosphate 2: Iron only: iron tablets containing 5 mg of ferric pyrophosphate (1.5 mg as iron) and 1.5 g of carbohydrate 4 × /day, 8 weeks	Hb: ∅^1 2 a^ Ferritin: ∅^1 a^ ↓^2^ SI: ∅^1 a^ ↓^2^	
Matter et al. 1987 [59]	Nonrandomized experimental study *n* = 27	Running (marathon) NR: reproductive health history included	1: No treatment 2: 500 mg of amino acid chelate iron 3: 5 mg of folic acid 4: 60 mg of lactose sugar placebo 1 × /day, 10 weeks	Hb: NR^1 4^ ∅^2 3^ Ferritin: NR^1 3^ ↑^2^ ↓^4^ SI: NR^1 3^ ∅^2^ ↓^4^	VO_2_max: NR^1^ ∅^2 3 4^ Lactate: ∅^1 2 3 4 a^
McCormick et al. 2020 [60]	Nonrandomized experimental study *n* = 9	Running 27 ± 6 years	1: 325 mg of ferrous sulfate and 500 mg of ascorbic acid 1 × /day, 8 weeks	Ferritin: ↑^1^	
Pitsis et al. 2004 [61]	Nonrandomized experimental study *n* = 36	Mixed elite level sports 18 years	1: 325 mg ferrous sulfate plus 500-mg tablet of vitamin C 1 × /day, 60 days	Hb: ∅^1^ Ferritin: ↑^1^ HCT: ∅^1^ SI: ∅^1^	
Plowman and McSwegin 1981 [62]	Nonrandomized experimental study *n* = 32	Running (cross country)	1: Control no treatment 2: 500 mg ascorbic acid 3: 1170 mg ferrous sulfate + 450 mg ascorbic acid 1 × /day, 12 weeks	Hb: ∅^1 2^, ↑^3 a^ SI: ∅^1 2 3 a^	
Sandroni et al. 2022 [63]	RCT *n* = 19	Mixed DIII Collegiate sports 19.9 ± 1.6 years	1: Single-serve packet of synbiotic supplement with 5 g prebiotic fiber + 8 billion CFU probiotic *B. lactis* + 8 mg of Fe per day as ferrous sulfate tablets 2: Placebo packet with 5 g maltodextrin powder + 8 mg of Fe per day as ferrous sulfate tablets 1 × /day, 8 weeks	Hb: ∅^1 2 a^ Ferritin: ↑^1 a^, ∅^2^	
Yoshida et al. 1990 [64]	RCT *n* = 12	Running Arm 1: 19.7 ± 1.1 years Arm 2: 19.5 ± 1.1 years	1: 200 mg ferrous citrate sodium succinate (20 mg elemental iron) with saccharide, vitamins C, B_2_, B_6_, and folic acid 2: Placebo tablets 3 × /day, 8 weeks	Hb: ∅^1 2 a^ Ferritin: ↑^1 a^, ↓^2^ HCT: ∅^1 2 a^	VO_2_max: ∅^1 2 a^

*RCT* randomized controlled trial, *Hb* hemoglobin*, HCT* hematocrit*, SI* serum iron*, CRP* C-reactive protein*, IL-6* interleukin 6, *CFU* colony-forming units, *NR* not reported, *SEM* standard error of the mean

↑ Statistically significant increase (*p* ≤ 0.05)

↓ Statistically significant decrease (*p* ≤ 0.05)

∅ No statistically significant difference

^1^ = arm 1, ^2^ = arm 2, ^3^ = arm 3, ^4^ = arm 4, ^a^ = intervention versus control

#### Participant Characteristics

Only a few studies included reproductive health status or contraceptive use. The participant age range was 12–45 years old. Some studies recruited participants according to baseline iron status [30, 38, 59], while other studies stratified the analysis based on baseline iron status after the intervention [34].

#### Effect of Oral Iron Supplementation on Iron Status

Below, we have grouped oral iron supplements into iron salts, chelated iron, heme iron, and other unknown forms of iron. The iron salt interventions were the most common and included ferrous sulfate (*n* = 11) [24–34].

##### Ferrous Sulfate

In total, 11 studies investigated the effect of ferrous sulfate supplementation on athletes’ iron status, with mixed but generally favorable outcomes for iron biomarkers. Dosages ranged from 100 to 975 mg daily, with intervention durations between 2 and 13 weeks. Significant improvements in ferritin and/or Hb were observed in several trials, particularly when participants had low baseline iron stores. For example, Brigham et al. [24], Fogelholm et al. [26], Schoene et al. [34], and LaManca et al. [28] reported significant increases in Hb and/or ferritin in the ferrous sulfate group. The placebo groups experienced declines or no change.

In contrast, other studies found no significant effects, such as those by Dellavalle et al. [25], McClung et al. [29], Pate et al. [31], and Powell et al. [32], where no statistically significant changes in iron biomarkers were observed within or between groups following ferrous sulfate supplementation. Across studies, performance outcomes like VO_2_max and lactate remained largely unaffected, even when iron biomarkers improved. These results suggest that ferrous sulfate can improve iron status, especially in iron-deficient athletes, but its impact on VO_2_max is less pronounced. Baseline iron status, training regimen, dose, and supplementation duration likely influence outcomes.

##### Other Iron Salts

The remaining trials used ferrous pyrophosphate, gluconate, and fumarate. Intervention durations varied from 3 to 13 weeks. The impact on iron biomarkers was variable in these trials. For example, Karamizrak et al. [37] and Flynn et al. [35] reported statistically significant increases in some markers of iron status. In contrast, Kapoor et al. [36] and Booth et al. [23] reported no changes in iron status after supplementation. None of these studies measured VO_2_max.

##### Chelated Iron

The chelated iron used in this trial is iron bound to two glycine molecules. Iron in this form is thought to be more easily absorbed with fewer gastrointestinal side effects than traditional iron salts [65]. Schulte et al. [38] investigated the impact of 25 mg of elemental iron as ferrous bisglycinate (Ferrochel chelate) in 33 track and field and soccer players with iron deficiency (nonanemic). There was no change in Hb, and both groups showed a statistically significant increase in ferritin from baseline to post intervention.

##### Heme Iron

Miller et al. [39] compared the effects of 600 mg of bovine heme iron versus a placebo once daily for 8 weeks among 21 runners. The iron group had a statistically significant decrease in Hb, and the placebo group did not.

##### Unspecified Forms of Iron

Kang et al. [40] randomized 25 soccer players to a 15-ml solution containing 40 mg of elemental iron or a 15-ml control solution. The iron intervention group had no change in Hb, whereas the control group had a statistically significant decrease in Hb. There was a statistically significant increase in ferritin in the iron supplement group but no change in the control group. Both groups reported a statistically significant decrease in HCT.

#### Effect of Other Oral Supplements on Iron Status

Five studies tested other oral supplements’ effects on athletes’ iron status [41–45]. Cialdella-Kam et al. [41] conducted a nonrandomized single-arm trial in which eight endurance athletes added and consumed 325 mL of Gatorade^®^ Nutrition Drink (54 g carbohydrates, 20 g protein) in their daily diet for 6 months. No significant changes in ferritin, serum iron, or VO_2_max were observed. The other four studies were RCTs. Interventions including bovine colostrum, pea powder, and soy protein powder were unsuccessful in improving iron status [42, 44, 45]. Interestingly, a pairwise comparison revealed that IL-6 levels were significantly higher in the control group compared with the bovine colostrum group after 6 months of supplementation [42]. Sha et al. [43] randomized 20 soccer players to 1.5–2 L of hydrogen-enriched or control water, consistent with the participants’ previous habits over 8 weeks. While there were no between-group differences, there was a statistically significant increase in Hb in the hydrogen-rich water group post intervention. In addition, IL-6 was significantly lower in the enriched water group, suggesting a potential anti-inflammatory effect of hydrogen-rich water. No statistically significant changes were observed in studies that measured C-reactive protein (CRP) or VO_2_ max [41, 44].

#### Effect of Food on Iron Status

There were five food-based interventions [46–50]. Three were single-arm trials, and two were RCTs. Alaunyte et al. [46], Łagowska et al. [49], and Mugambi et al. [50] provided various iron-rich foods for 6–12 weeks. All three studies reported favorable outcomes for markers of iron status, although only Łagowska et al. [49] and Mugambi et al. [50] reached statistical significance. Hennigar et al. [47] and Karl et al. [48] conducted RCTs among US female Army members undergoing basic combat training. Both studies provided food bars to participants with varying nutrient profiles. One group receiving an iron-fortified food bar reported significantly higher Hb levels compared with placebo post intervention.

#### Effect of Dietary Lifestyle Change on Iron Status

Three single-arm studies and one RCT examined the impact of dietary lifestyle change on iron-related biomarkers [51–55]. Durkalec-Michalski et al. [51] assigned 11 CrossFit athletes to a ketogenic diet for 4 weeks, defined as ≥ 75% daily energy from fat, 1.7 g of protein per kilogram of body mass, and up to 5% energy from carbohydrates. Body composition and energy expenditure measurements were used to individualize calorie recommendations for weight maintenance. Participants were also provided a 10-day menu, food diary, and kitchen scale. The results showed a statistically significant decline in Hb. There were no substantial changes in HCT or VO_2_max. Graja et al. [52] recorded no significant change in Hb or HCT among 12 handball players after 1 month of intermittent fasting during Ramadan. An iron-rich diet prescription, performance nutrition counseling, a fixed menu, and nutrition education were among the interventions that successfully improved markers of iron status [53–55].

#### Combination Therapies

Nine studies implemented a combination of interventions defined as an intervention that used an oral iron supplement in at least one group and incorporated an additional dietary component, either administered alongside the iron (e.g., vitamin C or milk) or used as a comparator in another study arm [56–64]. In total, five RCTs and four non-RCTs were included. All combination therapies, except in Koikawa et al. [58], reported favorable outcomes for iron status. In studies that measured performance or inflammatory markers, no significant changes were observed [56, 59, 64].

### Meta-Analysis

#### Effects of Ferrous Sulfate Supplement on Iron Status

##### Hemoglobin

**Fig. 2:**
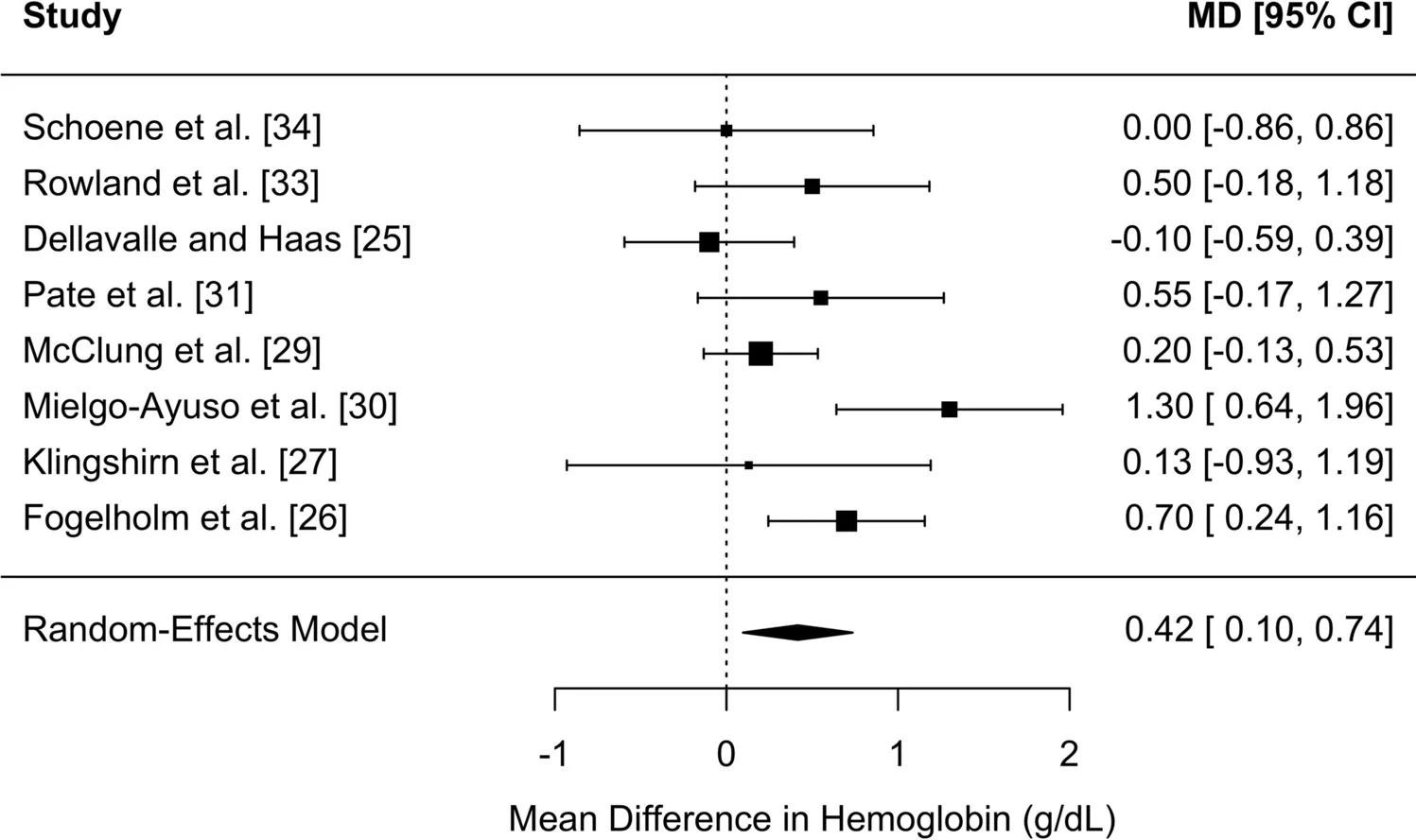
Random effects model: impact of ferrous sulfate supplementation on hemoglobin

Eight RCTs testing ferrous sulfate and with post-intervention Hb concentrations were included in the meta-analysis [25–27, 29–31, 33, 34]. The pooled estimate indicated a statistically significant increase in Hb associated with oral ferrous sulfate supplementation, with a mean difference of 0.42 g/dL (95% confidence interval [CI]: 0.10 to 0.74, *p* = 0.0109), favoring the intervention group (Fig. 2). Between-study heterogeneity was moderate, with *I*^2^ = 56.6%, *τ*^2^ = 0.11, and Cochran’s *Q* = 15.42 (*p* = 0.031), suggesting moderate variability not attributable to sampling error alone. Influence diagnostics identified Mielgo-Ayuso et al. [30] as moderately influential, with elevated Cook’s distance and strong influence on between-study heterogeneity. However, leave-one-out sensitivity analysis confirmed the robustness of the findings. These results suggest that iron supplementation with ferrous sulfate in active females leads to a modest but statistically significant improvement in Hb compared with control, with acceptable heterogeneity across studies.

##### Ferritin

**Fig. 3:**
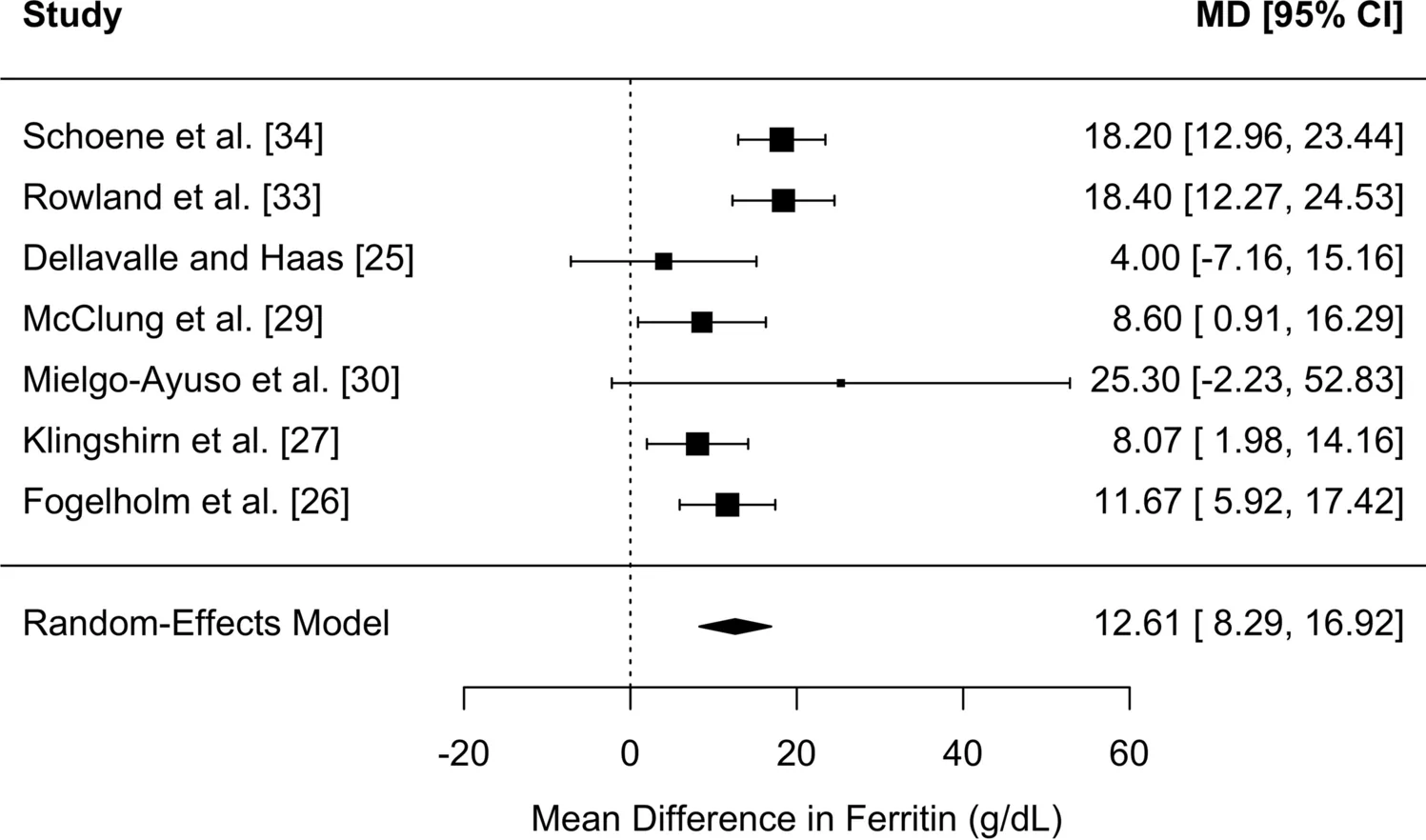
Random effects model: impact of ferrous sulfate supplementation on ferritin

Seven RCTs intervening with ferrous sulfate reported post-intervention ferritin concentrations [25–27, 29, 30, 33, 34]. The pooled analysis revealed a statistically significant increase in ferritin following iron supplementation compared with the control group, with a mean difference of 12.61 ng/mL (95% CI: 8.29–16.92, *p* < 0.0001) (Fig. 3). Heterogeneity was moderate, with *I*^2^ = 57.38% and *τ*^2^ = 17.66, and the *Q*-test indicated statistically significant heterogeneity (*Q* = 13.99, *p* = 0.0297). Sensitivity analyses using a leave-one-out approach showed that the pooled estimate remained statistically significant across all iterations, with mean differences ranging from 11.28 to 13.6 ng/mL, indicating that no single study unduly influenced the overall result.

#### Ferritin and Hemoglobin Subgroup Analysis

**Fig. 4:**
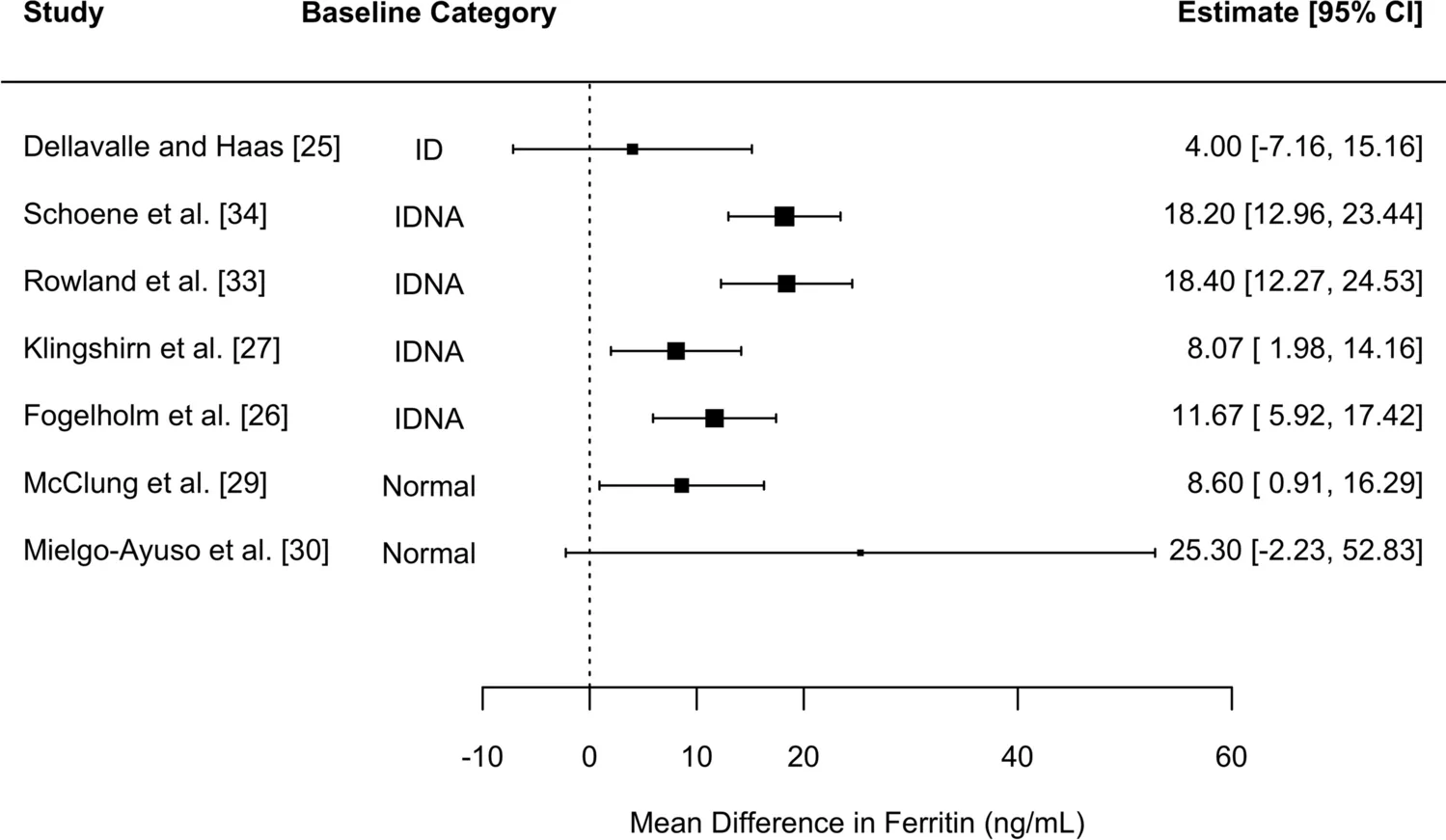
Subgroup analysis: Ferritin stratified by baseline iron status. ID iron deficiency stage 1, IDNA iron deficiency stage 2

For subgroup analysis, the baseline iron status of each study population was categorized as follows: normal (mean ferritin > 35 ng/mL), iron deficiency stage 1 (ID) (mean ferritin < 35 ng/mL), and iron deficiency without anemia stage 2 (IDNA) (mean ferritin < 20 ng/mL) [66]. When stratified by baseline iron status, greater increases in ferritin concentrations were observed in participants with IDNA compared with those with ID. The IDNA subgroup reported a statistically significant pooled mean difference of 14.16 ng/mL (95% CI: 9.22–19.10, *p* < 0.0001). In contrast, the single study in the ID subgroup yielded a nonsignificant estimate, despite a positive direction of effect (Fig. 4). Among studies with overall normal iron status, effects were mixed, with one significant improvement [29] and one with wide uncertainty [30] likely owing to variability in baseline stores or dose–response sensitivity.

**Fig. 5:**
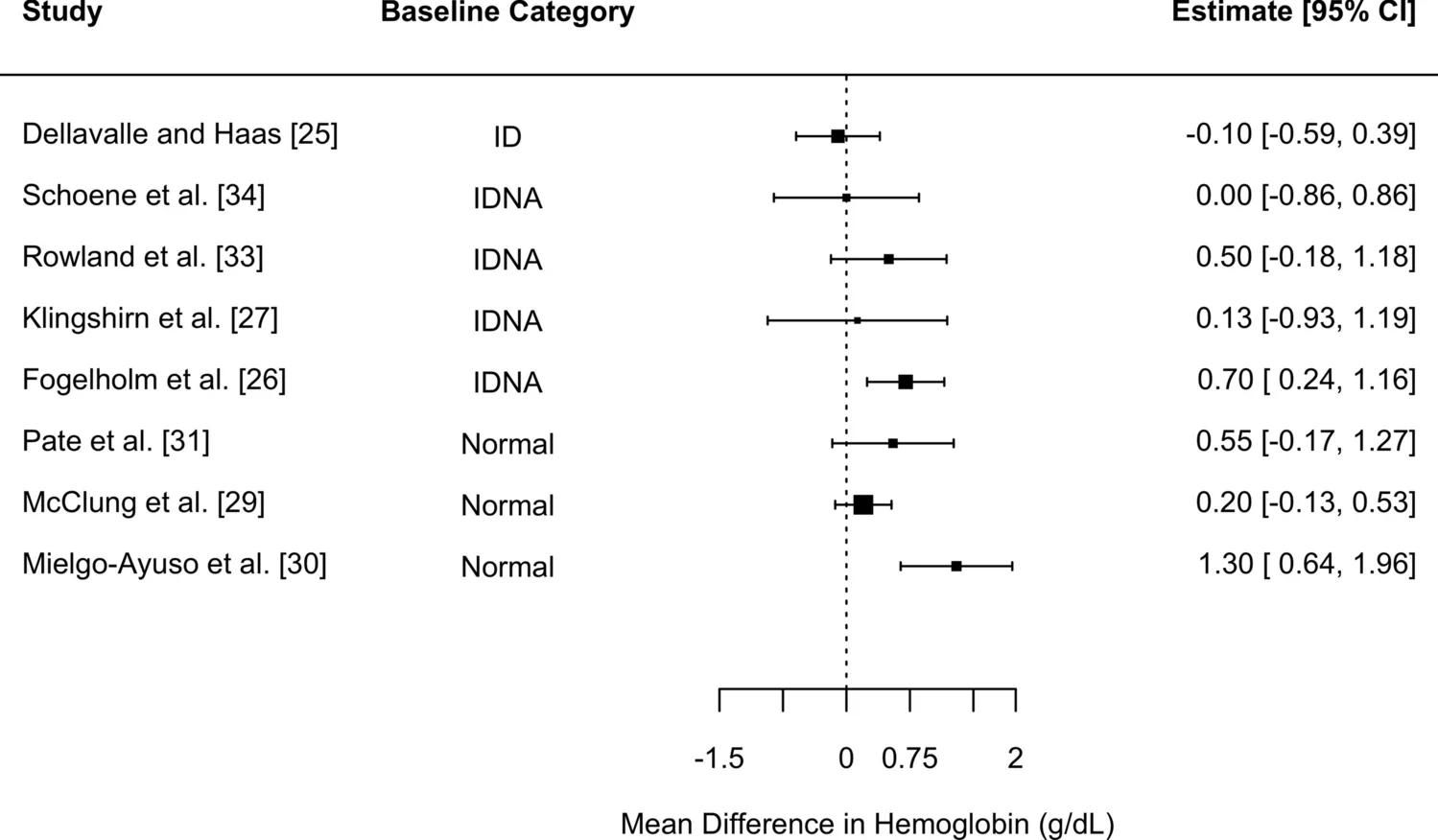
Subgroup analysis: hemoglobin stratified by baseline iron status. ID iron deficiency stage 1, IDNA iron deficiency stage 2

When stratified by baseline iron status, greater increases in Hb concentrations were observed in participants with IDNA compared with those with ID. The IDNA subgroup reported a statistically significant pooled mean difference of 0.49 g/dL (95% CI: 0.1651–0.8244, *p* = 0.0033). In contrast, the ID and normal subgroups yielded a nonsignificant estimate (Fig. 5).

#### Ferritin and Hemoglobin Meta-Regression: Dose

**Fig. 6:**
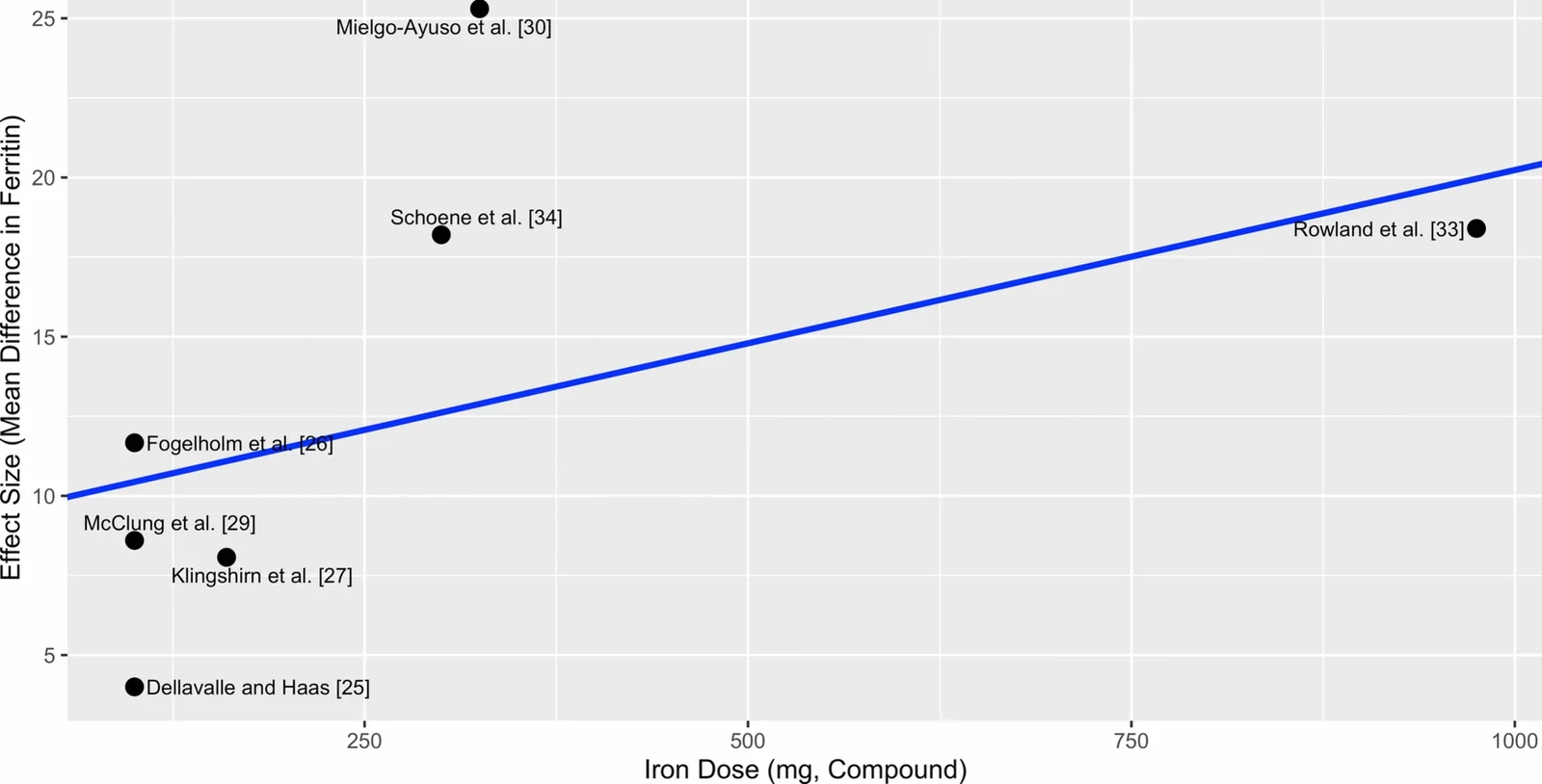
Relationship between ferrous sulfate dose and ferritin response

**Fig. 7:**
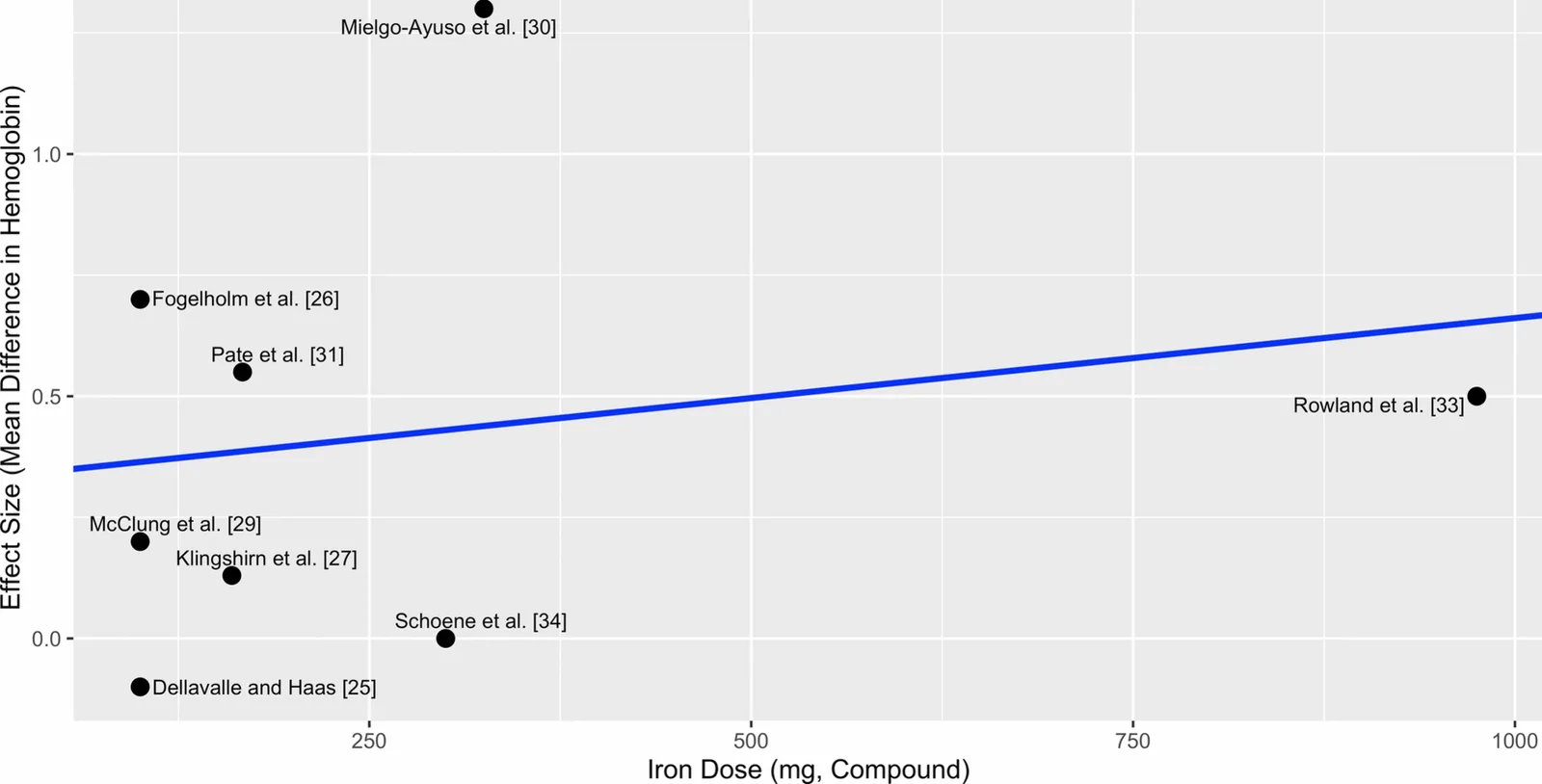
Relationship between ferrous sulfate dose and hemoglobin response

Meta-regression analysis revealed a positive association between ferrous sulfate dose and ferritin response but did not reach statistical significance (*β* = 0.0109 ng/mL per mg of iron; 95% CI: − 0.0004 to 0.0221; *p* = 0.0578), with dose accounting for 49.91% of the between-study heterogeneity. This suggests that higher doses of elemental iron were associated with greater increases in ferritin concentrations among active females (Fig. 6). Meta-regression analysis revealed a slight positive association between ferrous sulfate dose and Hb response but did not reach statistical significance (*β* = 0.0003 g/dL per mg of iron; 95% CI: − 0.0009 to 0.0016; *p* = 0.6). This suggests that higher doses of elemental iron were associated with very small increases in Hb concentrations among active females (Fig. 7).

#### Ferritin and Hemoglobin Meta-Regression: Duration

**Fig. 8:**
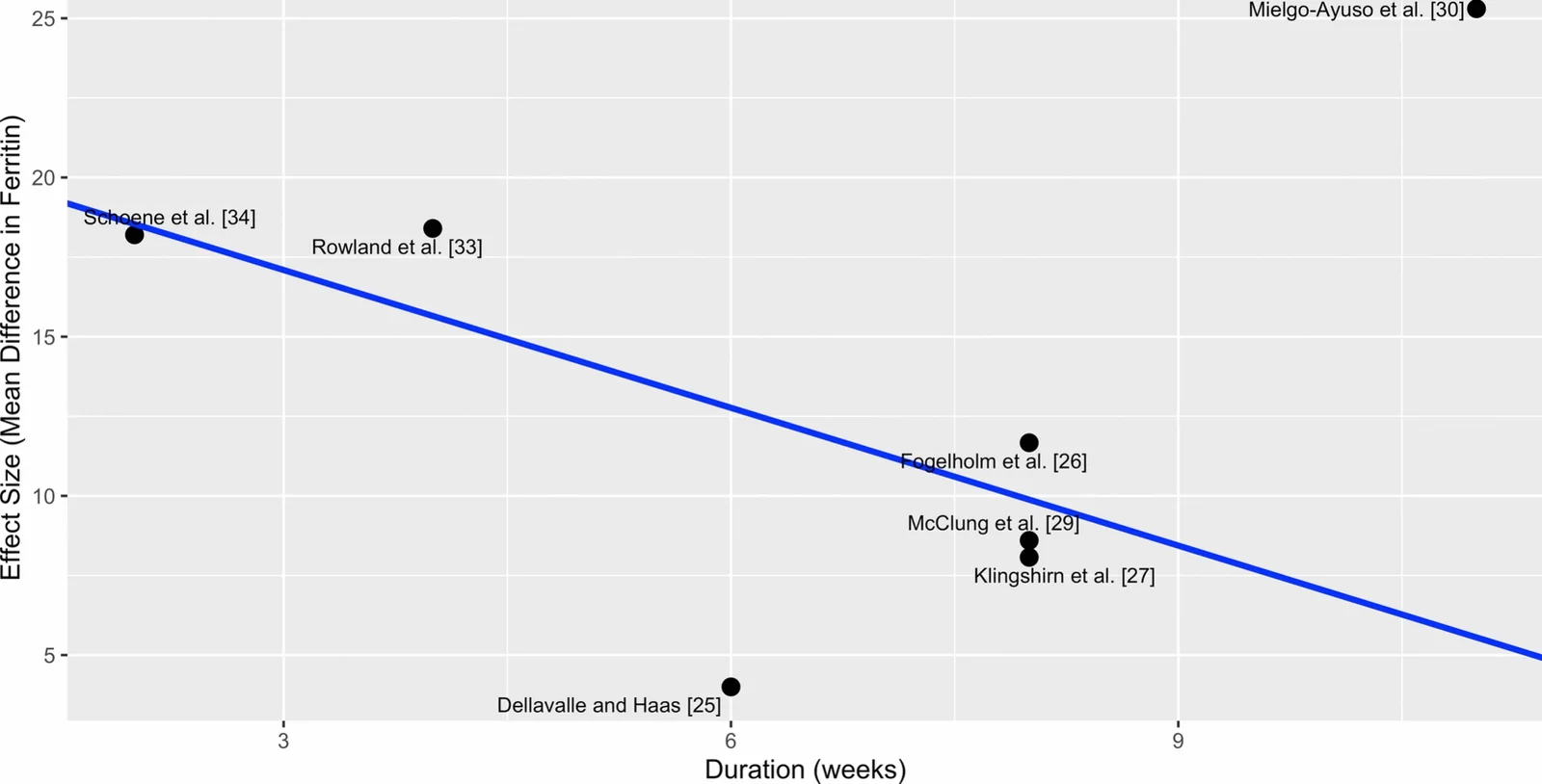
Relationship between ferrous sulfate duration and ferritin response

Meta-regression of ferritin also revealed intervention duration was a significant moderator (*β* = − 1.44 ng/mL per week, 95% CI: − 2.44 to − 0.45, *p* = 0.0046), indicating that longer interventions were associated with smaller increases in ferritin. The model explained 100% of between-study heterogeneity (*R*^2^ = 100%, *I*^2^ = 0%), suggesting duration as a key variable in differences across studies. The negative association may reflect variability in intervention adherence or “saturation point” in ferritin stores (Fig. 8).

**Fig. 9:**
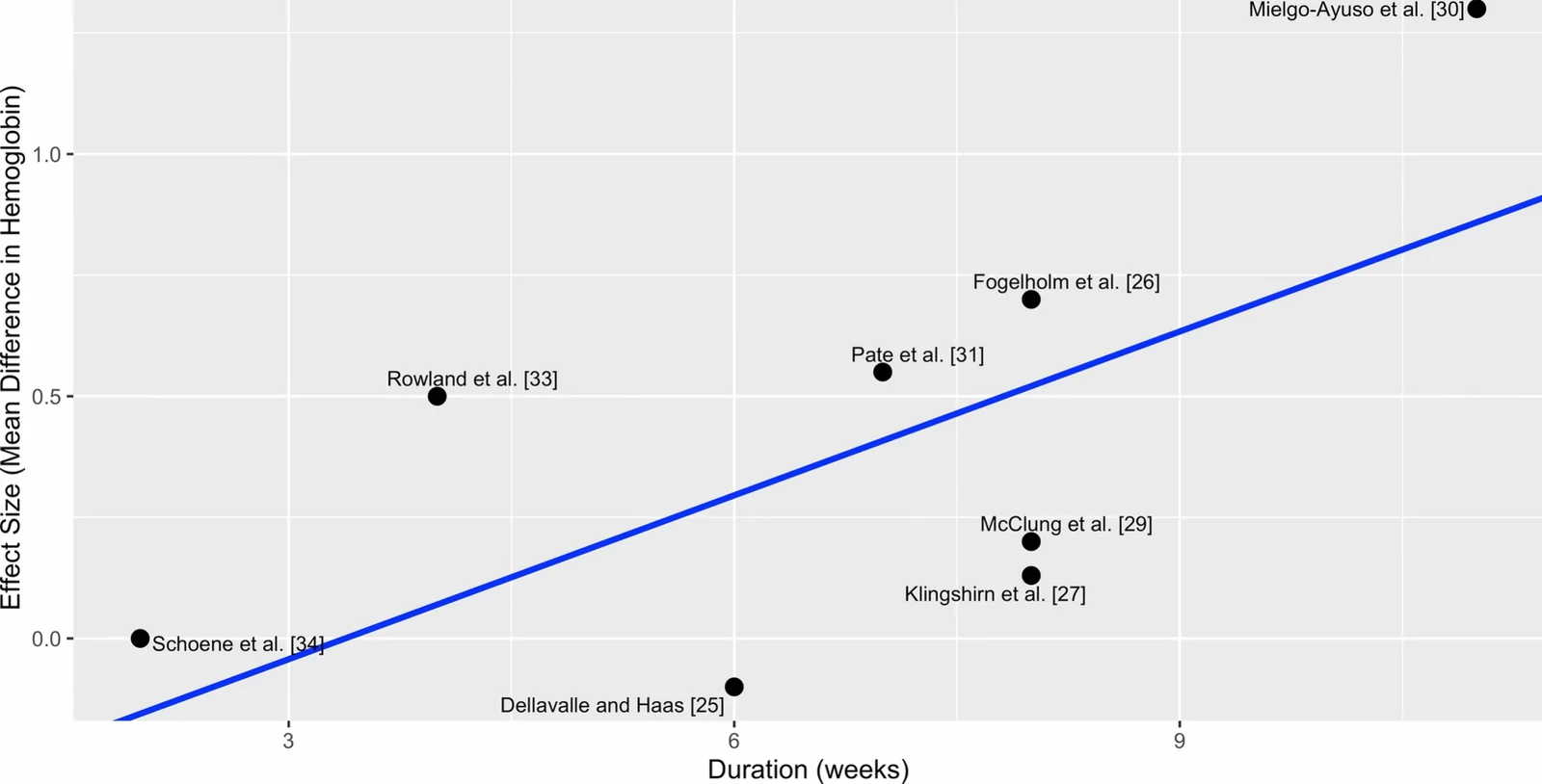
Relationship between ferrous sulfate duration and hemoglobin response

Meta-regression of Hb showed a positive trend; longer supplementation duration is associated with larger Hb gains (*β* = 0.11 g/dL per week, 95% CI: − 0.0074 to 0.2329, *p* = 0.0658). The model explained about 42% of the between-study variability (*R*^2^ = 42.1%), and the residual heterogeneity was moderate (*I*^2^ = 43.9%) (Fig. 9).

#### Ferritin and Hemoglobin Multivariable Random-Effects Meta-Regression

**Fig. 10:**
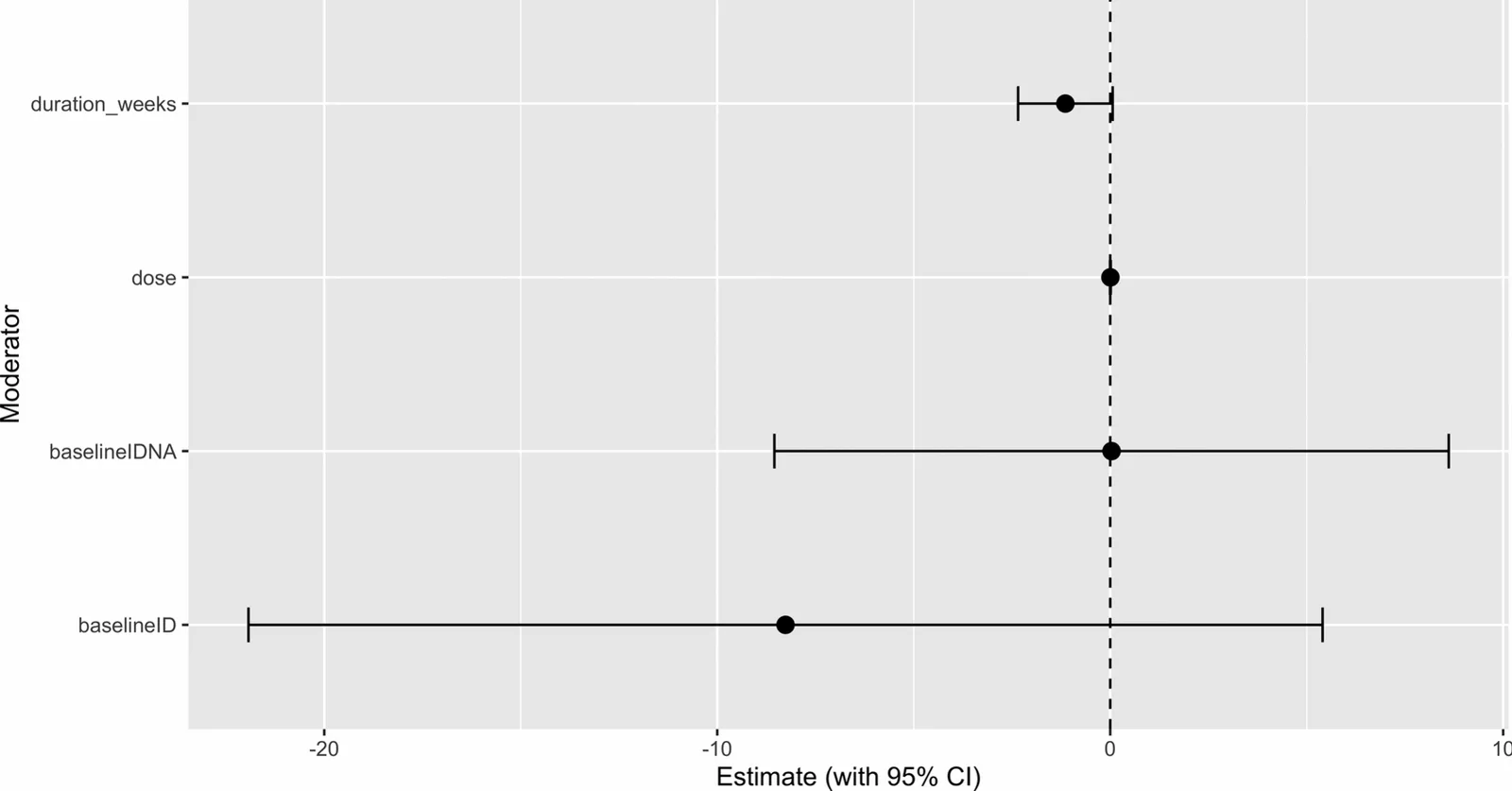
Multivariate random-effects meta-regression for ferritin outcome: dose, baseline iron, and duration

A multivariable random-effects meta-regression was conducted to examine whether iron dose (mg), baseline iron status (ID, IDNA, or normal), and intervention duration (weeks) moderated the effect of iron supplementation on changes in ferritin. The combination of predictors explained all between-study variability in ferritin outcomes (*R*^2^ = 100%, *I*^2^ = 0%). The overall model was statistically significant (*p* = 0.022). However, none of the individual covariates reached statistical significance. Duration exhibited a negative trend, suggesting that longer supplement duration may be associated with smaller ferritin gains (*β* = − 1.14 ng/mL per week, *p* = 0.063). Iron dose showed a small, positive association with ferritin (*β* = 0.005 ng/mL per mg of iron, *p* = 0.35). Compared with participants with normal baseline iron status, those with ID (*β* = − 8.26, *p* = 0.24) and IDNA (*β* = 0.03, *p* = 0.99) did not experience significantly different responses. Overall, the model explained additional heterogeneity but none of the covariates independently predicted ferritin response (Fig. 10).

**Fig. 11:**
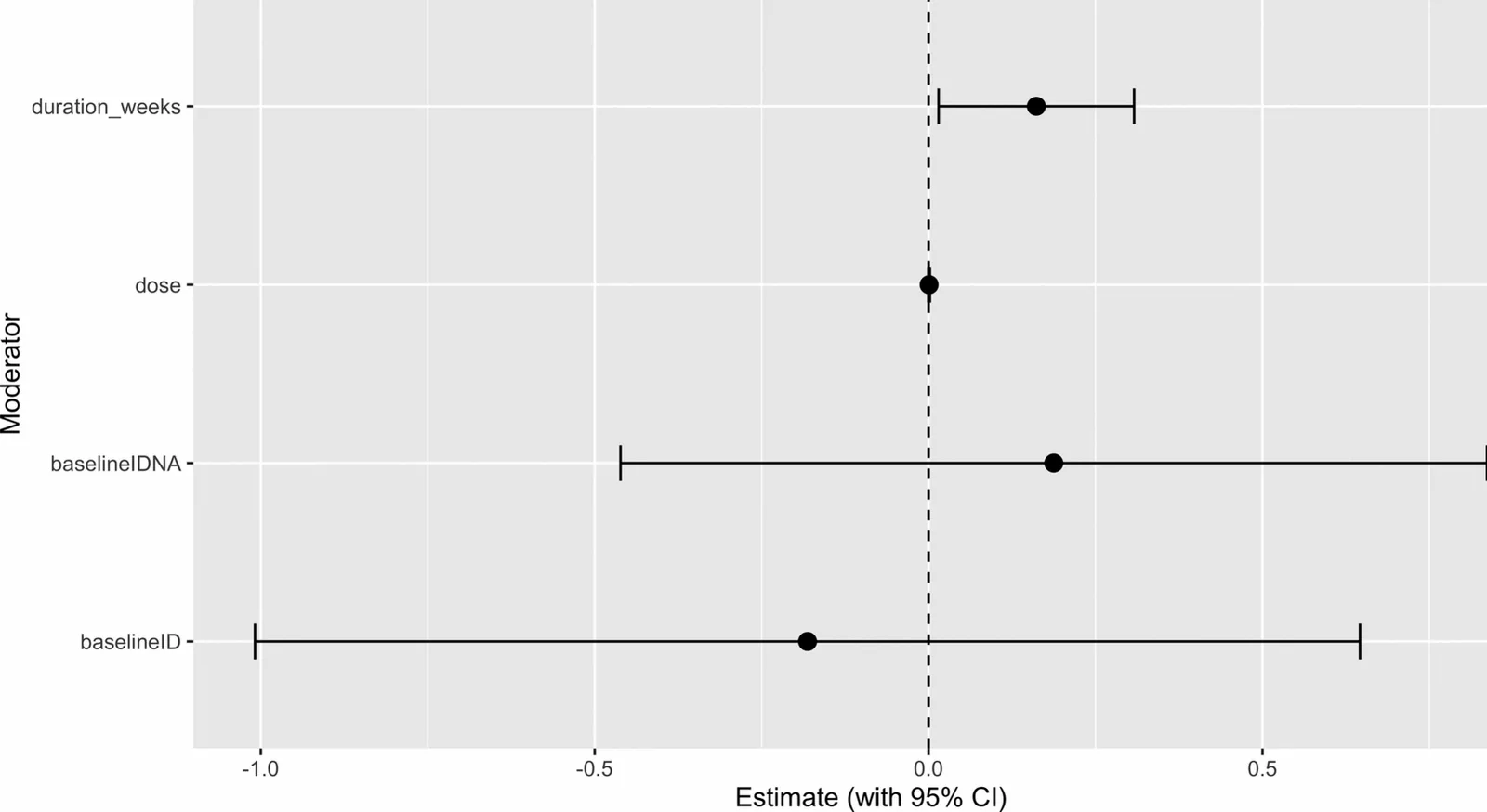
Multivariate random-effects meta-regression for hemoglobin outcome: dose, baseline iron, and duration

In the joint meta-regression model including dose, baseline iron status, and intervention duration, residual heterogeneity was low (*I*^2^ = 25%, *R*^2^ = 67%), and duration was the only independent predictor of hemoglobin change (*β* = 0.16 g/dL per week, 95% CI: 0.02 to 0.31, *p* = 0.031), while dose and baseline status were not significant (Fig. 11).

## Discussion

This systematic review summarizes the evidence from 42 trials examining the effect of diet and dietary supplements on iron status and performance of active females of reproductive age. Overall, findings suggest that oral iron supplementation and iron-rich food interventions will likely improve iron status. None of the 15 interventions that measured performance (VO_2_max or lactate) or inflammatory outcomes (hepcidin, CRP, or IL-6) produced statistically significant changes [26–28, 32, 34, 39, 41–44, 48, 51, 56, 59, 64].

Oral iron supplement interventions alone produce favorable outcomes in improving iron status. However, in some cases, oral iron supplementation did not raise mean ferritin in the intervention groups; it remained unchanged, while the placebo groups had a decrease in mean ferritin [23, 29, 30], suggesting that in some populations, an iron supplement may simply prevent a decline in iron status versus replenish an individual’s iron status. Interventions in the “other oral supplement” category were largely ineffective at improving iron status [41, 42, 44, 45], attributable to poor absorption, low iron content in the products used, or short study duration. The results from dietary lifestyle-change interventions were mixed, with the most successful intervention in this category involving nutrition education [54, 55]. Unsuccessful dietary lifestyle interventions such as the ketogenic diet [51] and intermittent fasting [52] may be, in some cases, counterproductive to maintaining iron status owing to decreases in carbohydrate or overall energy intake. Food-based strategies had some success, particularly iron-rich food interventions that included iron-fortified food bars [48], iron-rich meals [49], and “Athle-food”, an iron-rich porridge [50]. Studies involving combination therapies (e.g., oral iron supplement with additional dietary component) were also largely successful, with certain additions, such as ascorbic acid [60–62, 64], probiotic [56], or prebiotic [63] supplementation providing added benefit compared with OIS alone, likely owing to their effects on improving iron absorption in addition to high iron content.

Meta-analysis evaluated the effects of oral ferrous sulfate supplementation on hematological outcomes in active females. Pooled results across eight RCTs showed a statistically significant increase in Hb with moderate between-study heterogeneity. In the seven RCTs evaluating ferritin responses, a similar pattern emerged: supplementation led to a significant pooled increase in ferritin, also with moderate heterogeneity. When stratified by baseline iron status, the ferritin response was the largest in participants with IDNA stage 2. In contrast, changes in ferritin were nonsignificant in participants with ID stage 1 or normal iron status. This supports the notion that initial iron stores may influence response magnitude, though statistical power was limited in stratified analyses. Meta-regression also revealed that a greater iron dose is associated with a greater ferritin response. Interestingly, meta-regression showed a positive trend between Hb effect size and intervention duration and a negative trend between ferritin effect size and intervention duration. This could be representative of iron storage repletion, meaning the size of ferritin gains declines over time because ferritin reaches a “saturation point.” The multivariable meta-regression model showed that neither baseline iron status, dose, nor duration independently predicted ferritin response. However, duration did independently predict Hb response in this joint model.

While the studies in this review provide valuable insights, several limitations and study variations should be considered when interpreting the results.

### Study Duration and Sample Size

Short study periods and small sample sizes were noteworthy limitations to many included trials. A total of 36 of the 42 included articles had a sample size of less than 50 participants, potentially lacking statistical power. Furthermore, study duration varied greatly, with the shortest study lasting 2 weeks and the longest 6 months (26 weeks). Collectively, 34 of the studies included in this review lasted less than 12 weeks. Study duration is particularly relevant in tracking hematologic variables owing to the time needed for red blood cell turnover, iron recycling, and storage [67, 68]. The lifespan of a red blood cell is about 120 days (7 weeks), and in cases of iron deficiency anemia, Hb levels may begin to improve after 2 weeks of traditional iron supplementation. However, body iron stores need closer to 12 weeks to replenish completely [68].

### Reproductive-Health History

Reproductive-health history was seldom collected, yet it can provide a critical context for understanding an individual’s iron status. For example, contraceptive use can increase or decrease iron loss through menstruation [69, 70], making it essential to account for reproductive health in studies on female athletes.

### Dietary Intake and Role of Vitamin C

Another limitation was the lack of dietary intake data in many studies, particularly regarding total iron intake. The studies sometimes relied on self-reported dietary data, which can introduce bias or inaccuracies. In addition, vitamin C plays a well-documented role in enhancing the absorption of nonheme iron, the form of iron typically found in plant-based foods and supplements [71, 72]. This is particularly important in iron deficiency, as increasing absorption can improve iron status. Some studies included in the review administered vitamin C as part of the intervention [60–62, 64]. In contrast, others simply instructed participants to consume vitamin C-rich foods alongside their iron supplementation [26]. In these cases, the effect of vitamin C was not directly controlled for but could still have influenced the results. This discrepancy in how vitamin C was incorporated into the interventions could introduce outcome variability. For example, studies that did not account for vitamin C intake might underestimate the effectiveness of iron supplementation or fail to identify the role that vitamin C plays in enhancing iron absorption. However, studies that actively included vitamin C as part of the intervention could show pronounced improvement in iron status, making it difficult to attribute improvements solely to the iron supplement itself.

### Baseline Iron Status

Iron homeostasis is a balancing act, as both deficiencies and surplus of iron can be harmful. Therefore, an individual’s baseline iron status indicates how they might respond to iron-targeting interventions [73]. Athletes with pre-existing iron deficiency are more likely to respond to diet or supplement interventions. In contrast, those with sufficient iron stores might not experience significant changes in markers of iron status. Some studies recruited participants according to baseline iron status [30, 38, 59], while other studies stratified the analysis based on baseline iron status after the intervention [34]. In contrast, others may not have adequately accounted for this factor. Therefore, failing to control for baseline iron status could make it appear that the intervention had a greater or lesser effect than it did. Future research could provide clearer insights into how different interventions work across various baseline iron status conditions by stratifying participants.

### Activity Variability

Although our inclusion criteria required participants to train at least three times per week and identify with a specific sport or activity [18], the study populations included in the review varied in terms of both activity level and type. In addition, some studies introduced new training protocols, which may have contributed to fluctuations in iron status and responses to the intervention. Therefore, the variability in activity levels, even within an athletic population, represents a limitation of this review.

### Supplement Timing

Supplement timing is another factor that may influence iron absorption yet it was seldom discussed in the articles reviewed. Hepcidin exhibits diurnal variation, with the lowest levels in the morning [74]. This variation, combined with the inflammatory response to exercise, suggests that morning or within 30 min of morning exercise, before the inflammatory response to exercise is realized, may be ideal for iron supplement ingestion to avoid blunted absorption owing to increased hepcidin [68, 74].

### Clinical Relevance

Our meta-analysis revealed that ferrous sulfate supplementation increased ferritin concentration by an average of 12.61 ng/mL. This change is clinically relevant and may move athletes with low baseline iron status across iron-deficiency stages. The magnitude of change in hemoglobin was more modest, with an increase of 0.42 g/dL, which may be a result of the short duration of many of the interventions. These findings suggest that ferrous sulfate supplementation is an effective strategy to improve iron stores among active females.

Most studies that measured performance outcomes reported insignificant changes in VO_2_max and lactate after diet or dietary supplement intervention [26–28, 32, 39, 41, 44, 51, 56, 59, 64]. This could be representative of training adaptation and the baseline severity of iron deficiency. The average baseline iron status in the populations evaluated in this review may not have been low enough to result in a noticeable improvement in performance despite improvements in iron status post intervention.

## Conclusion

This systematic review provides an extensive overview of diet and dietary supplement interventions and their impact on iron status in active females. Our meta-analysis revealed that ferrous sulfate supplementation significantly improved Hb and ferritin levels in active females. The effects were most pronounced in those with low baseline iron stores in stratified analyses. Dose showed a positive trend, and duration showed a negative trend, toward modifying ferritin response. In the joint model including baseline iron status, dose, and duration, no single factor consistently predicted ferritin response; however, duration did independently predict Hb response. Outside of the studies included in the meta-analysis, OIS with or without additional dietary components, such as ascorbic acid, produced the most favorable outcomes. Iron-rich food-based interventions were also successful in improving iron status. Future research should address limitations by incorporating larger sample sizes, longer study durations, and standardized protocols for supplementation. In addition, collecting comprehensive reproductive health data, activity levels, and factors like vitamin C intake and inflammation will enhance our understanding and ability to optimize iron interventions for female athletes. Given the variables discussed, personalized care remains important when preventing or treating iron deficiency in active females.

## Supplementary Information

Below is the link to the electronic supplementary material.

Supplementary file1 (PDF 56 KB)

## Declarations

### Data Availability

Data are available upon request.
