# Supplementary material for: The Effect of Diet and Dietary Supplements on Iron Status of Active Females: A Systematic Review and Meta-analysis of Interventional Trials
Source: Sports Med. 2026 Jun 10;56(10):2527–49. doi: 10.1007/s40279-026-02464-x (PMC13614990; doi:10.1007/s40279-026-02464-x)
Supplement: Supplementary file 1 [file 40279_2026_2464_MOESM1_ESM.pdf]

The Effect of Diet and Dietary Supplements on Iron Status of Female Athletes: A Systematic Review and Meta-analysis of Interventional Trials

Emma E. McCarthy[1] Josh P. Pekler[1], Tina Tran[2], Lauren E. Deluca[1], Jarrad Hampton-Marcell[3], Beatriz Penalver Bernabe[4], Karrie Hamstra-Wright[1], Mary Dawn Koenig[5], Danielle Colegrove[6], Lisa Tussing-Humphreys[1]

[1]Department of Kinesiology and Nutrition, University of Illinois Chicago, Chicago, IL 60612, United States; [2]College of Osteopathic Medicine, Kansas City University, Joplin, MO 64804, United States; [3]Department of Biological Sciences, University of Illinois Chicago, Chicago, IL 60612, United States; [4]Department of Biomedical Engineering, University of Illinois Chicago, Chicago, IL 60612, United States; [5]Department of Human Development Nursing Science, University of Illinois Chicago, Chicago, IL 60612, United States, [6]Department of Athletic Medicine, University of Illinois Chicago, Chicago, IL 60612, United States

Corresponding author:

Lisa Tussing-Humphreys, RD, PhD

Professor

Department of Kinesiology and Nutrition, University of Illinois Chicago, 1919 West Taylor Street, Chicago IL, 60612

Tel: 312-355-5521, Email: tussing@uic.edu

Supplementary Materials

Supplementary Materials 1. Search Terms

PubMed Search Terms:

("iron deficiencies"[mesh] OR "iron deficienc*"[tiab] OR "iron deficiency anemia"[tiab] OR "iron homeostasis"[tiab] OR "iron status"[tiab] OR "iron deficient non anemia"[tiab] OR "ferritin"[tiab] OR "hepcidin"[tiab] OR "hemoglobin"[tiab] OR "anemi*"[tiab])

AND ("athletes"[mesh] OR "sports"[mesh] OR "athletic performance"[mesh] OR "exercise"[mesh] OR "relative energy deficiency in sport"[mesh] OR "athlete"[tiab] OR "athletes"[tiab] OR "collegiate"[tiab] OR "recreational"[tiab] OR "endurance"[tiab] OR "physical performance"[tiab] OR "military"[tiab] OR "soldier"[tiab] OR "soldiers"[tiab])

AND ("diet therapy"[mesh] OR "diet, food, and nutrition"[mesh] OR "dietary iron"[tiab] OR "iron supplement"[tiab] OR "probiotic"[tiab] OR "probiotics"[tiab] OR "prebiotic"[tiab] OR "prebiotics"[tiab] OR "synbiotic"[tiab] OR "synbiotics"[tiab] OR "Lactobacillus plantarum 299v"[tiab] OR "heme"[tiab] OR "non-heme"[tiab] OR "carbohydrate"[tiab] OR "carbohydrates"[tiab] OR "fiber"[tiab] OR "vitamin c"[tiab] OR "iron-rich food"[tiab] OR "iron-fortified"[tiab] OR "anti-inflammatory"[tiab] OR "energy availability"[tiab] OR "dietary intervention*"[tiab] OR "dietary supplement*"[tiab])

Rehabilitation & Sports Medicine Source Search Terms:

(DE "IRON deficiency anemia" OR DE "IRON in the body" OR DE "FERRITIN" OR DE "HEMOGLOBINS" OR DE "TRANSFERRIN" OR AB “iron deficienc*” OR AB “anemi*” OR AB “iron homeostasis” OR AB “iron status” OR AB “iron deficient non anemia” OR AB “ferritin” OR AB “hepcidin” OR AB “hemoglobin” OR TI “iron deficienc*” OR TI “iron deficiency anemia” OR TI “iron homeostasis” OR TI “iron status” OR TI “iron deficient non anemia” OR TI “ferritin” OR TI “hepcidin” OR TI “hemoglobin” OR TI anemi*) AND (DE "ATHLETES" OR DE "ABORIGINAL Australian athletes" OR DE "AFRICAN athletes" OR DE "AMATEUR athletes" OR DE "ARAB athletes" OR DE "ARCHERS" OR DE "ASIAN athletes" OR DE "ATHLETES as actors" OR DE "ATHLETES in art" OR DE "ATHLETES with diabetes" OR DE "ATHLETES with disabilities" OR DE "BADMINTON players" OR DE "BASEBALL players" OR DE "BASKETBALL players" OR DE "BLACK athletes" OR DE "BOBSLEDDERS" OR DE "BODYBUILDERS" OR DE "BOWLERS" OR DE "BOXERS (Sports)" OR DE "BULLFIGHTERS" OR DE "CANADIAN athletes" OR DE "CANOEISTS" OR DE "CELEBRITY athletes" OR DE "CHILD athletes" OR DE "CHILDREN of athletes" OR DE "CHRISTIAN athletes" OR DE "COLLEGE athletes" OR DE "CRICKET players" OR DE "CROQUET players" OR DE "CURLERS (Athletes)" OR DE "CYCLISTS" OR DE "DEFENSIVE players" OR DE "ELITE athletes" OR DE "ENDURANCE athletes" OR DE "EUROPEAN athletes" OR DE "FENCERS" OR DE "FOOTBALL players" OR DE "GAY athletes" OR DE "GLADIATORS" OR DE "GOLFERS" OR DE "GYMNASTS" OR DE "HANDBALL players" OR DE "HIGH school athletes" OR DE "HOCKEY players" OR DE "INTERSEX athletes" OR DE "JAI alai players" OR DE "JEWISH athletes" OR DE "JUNIOR high school athletes" OR DE "KABADDI players" OR DE "LACROSSE players" OR DE "LAWN bowlers" OR DE "LGBTQ+ athletes" OR DE "LONG-term athlete development" OR DE "MALE athletes" OR DE "MARTIAL artists" OR DE "MEXICAN athletes" OR DE "MIDDLE school

athletes" OR DE "MOUNTAINEERS" OR DE "MUSLIM athletes" OR DE "NATIVE American athletes" OR DE "NETBALL players" OR DE "OFFENSIVE players" OR DE "OLDER athletes" OR DE "OLYMPIC athletes" OR DE "ORIENTEERS" OR DE "PACIFIC Islander athletes" OR DE "PROFESSIONAL athletes" OR DE "ROWERS" OR DE "RUGBY football players" OR DE "RUNNERS (Sports)" OR DE "SKATERS" OR DE "SKIERS" OR DE "SKYDIVERS" OR DE "SNOWBOARDERS" OR DE "SOCCER players" OR DE "SOFTBALL players" OR DE "SQUASH players" OR DE "STARTING players" OR DE "SUBSTITUTE players" OR DE "SURFERS" OR DE "SWIMMERS" OR DE "TABLE tennis players" OR DE "TEAM handball players" OR DE "TENNIS players" OR DE "TRACK & field athletes" OR DE "TRIATHLETES" OR DE "VOLLEYBALL players" OR DE "WATER polo players" OR DE "WEIGHT lifters" OR DE "WINDSURFERS (Persons)" OR DE "WOMEN athletes" OR DE "WRESTLERS" OR DE "RECREATION" OR DE "AERONAUTICAL sports" OR DE "AGE & sports" OR DE "AMATEUR sports" OR DE "BALL games" OR DE "BASEBALL" OR DE "COLLEGE sports" OR DE "COMBAT sports" OR DE "CONTACT sports" OR DE "CROSS-training (Sports)" OR DE "DOPING in sports" OR DE "ENDURANCE sports" OR DE "EXTREME sports" OR DE "GYMNASTICS" OR DE "HOCKEY" OR DE "INDIVIDUAL sports" OR DE "MILITARY sports" OR DE "MINORITIES in sports" OR DE "MOTORSPORTS" OR DE "OLYMPIC Games" OR DE "PROFESSIONAL sports" OR DE "RACKET games" OR DE "RECREATIONAL sports" OR DE "ROLLER skating" OR DE "SCHOOL sports" OR DE "SHOOTING (Sports)" OR DE "SKATEBOARDING" OR DE "SOFTBALL" OR DE "SPORT for all" OR DE "SPORTS for girls" OR DE "TEAM sports" OR DE "WINTER sports" OR DE "WOMEN'S sports" OR AB “athlete” OR AB “athletes” OR AB “collegiate” OR AB “recreational” OR AB “endurance” OR AB “physical performance” OR AB “military” OR AB “soldier” OR AB “soldiers” OR TI “athlete” OR TI “athletes” OR TI “collegiate” OR TI “recreational” OR TI “endurance” OR TI “physical performance” OR TI “military” OR TI “soldier” OR TI “soldiers”) AND (DE "DIETARY supplements" OR DE "PROBIOTICS" OR DE "PROTEIN powders" OR DE "SPORTS nutrition" OR DE "BODYBUILDER nutrition" OR DE "DIET in runners (Sports)" OR DE "NUTRITION" OR DE "ADOLESCENT nutrition" OR DE "AMINO acids in nutrition" OR DE "ANTIBIOTICS in nutrition" OR DE "CARBOHYDRATE drugs in sports" OR DE "CEREAL grasses in human nutrition"OR DE "CITRUS fruits in human nutrition" OR DE "DAIRY products in human nutrition" OR DE "DIETARY calcium" OR DE "DIETARY carbohydrates" OR DE "DIETARY fats" OR DE "DIETARY fiber" OR DE "DIETARY proteins" OR DE "DIETARY supplements"OR DE "ESSENTIAL fatty acids in human nutrition" OR DE "ESSENTIAL nutrients" OR DE "FISH oils in human nutrition" OR DE "FLAXSEED in human nutrition" OR DE "FOOD combining" OR DE "FRUCTOSE in human nutrition" OR DE "FRUIT in human nutrition" OR DE "LIPIDS in nutrition" OR DE "MICRONUTRIENTS" OR DE "MILK in human nutrition" OR DE "MINERALS in nutrition" OR DE "NUTRITIONAL requirements" OR DE "NUTRITIONAL value" OR DE "NUTRITIONISTS" OR DE "PHYSICAL fitness & nutrition" OR DE "RICE in human nutrition" OR DE "SNACK foods & health" OR DE "SUGARS in human nutrition" OR DE "UNSATURATED fatty acids in human nutrition" OR DE "VEGETABLES in human nutrition" OR DE "VITAMINS" OR DE "VITAMINS in human nutrition" OR AB “dietary iron” OR AB “iron supplement” OR AB “probiotic” OR AB “probiotics”OR AB “prebiotic” OR AB “prebiotics”OR AB “synbiotic” OR AB “synbiotics” OR AB “Lactobacillus

plantarum 299v” OR AB “heme” OR AB “non-heme” OR AB “carbohydrate” OR AB “carbohydrates” OR AB “fiber” OR AB “vitamin c” OR AB “iron-rich food” OR AB “iron-fortified” OR AB “anti-inflammatory” OR AB “energy availability” OR AB “dietary intervention*” OR AB “dietary supplement*” OR TI “dietary iron” OR TI “iron supplement” OR TI “probiotic” OR TI “probiotics” OR TI “prebiotic” TI “prebiotics” OR TI “synbiotic” OR TI “synbiotics”OR TI “Lactobacillus plantarum 299v” OR TI “heme” OR TI “non-heme” OR TI “carbohydrate” OR TI “carbohydrates”OR TI “fiber” OR TI “vitamin c” OR TI “iron-rich food” OR TI “iron-fortified” OR TI “anti-inflammatory” OR TI “energy availability” OR TI “dietary intervention*” OR TI “dietary supplement*”)

<u>Scopus Search Terms:</u>
(TITLE-ABS-KEY("iron deficienc*") OR TITLE-ABS-KEY("iron deficiency anemia") OR TITLE-ABS-KEY("iron homeostasis") OR TITLE-ABS-KEY("iron status") OR TITLE-ABS-KEY("iron deficient non anemia") OR TITLE-ABS-KEY("ferritin") OR TITLE-ABS-KEY("hepcidin") OR TITLE-ABS-KEY("hemoglobin") OR TITLE-ABS-KEY("anemi*"))

AND (TITLE-ABS-KEY("athlete") OR TITLE-ABS-KEY("athletes") OR TITLE-ABS-KEY("collegiate") OR TITLE-ABS-KEY("recreational") OR TITLE-ABS-KEY("endurance") OR TITLE-ABS-KEY("physical performance") OR TITLE-ABS-KEY("military") OR TITLE-ABS-KEY("soldier") OR TITLE-ABS-KEY("soldiers"))

AND (TITLE-ABS-KEY("dietary iron") OR TITLE-ABS-KEY("iron supplement") OR TITLE-ABS-KEY("probiotic") OR TITLE-ABS-KEY("prebiotic") OR TITLE-ABS-KEY("synbiotic") OR TITLE-ABS-KEY("Lactobacillus plantarum 299v") OR TITLE-ABS-KEY("heme") OR TITLE-ABS-KEY("non-heme") OR TITLE-ABS-KEY("carbohydrate") OR TITLE-ABS-KEY("carbohydrates") OR TITLE-ABS-KEY("fiber") OR TITLE-ABS-KEY("vitamin c") OR TITLE-ABS-KEY("iron-rich food") OR TITLE-ABS-KEY("iron-fortified") OR TITLE-ABS-KEY("anti-inflammatory") OR TITLE-ABS-KEY("energy availability") OR TITLE-ABS-KEY("dietary intervention*") OR TITLE-ABS-KEY("dietary supplement*"))

<u>CINAHL Search Terms:</u>
((MH "iron deficiencies+") OR AB("iron deficienc*") OR TI("iron deficienc*") OR AB("iron deficiency anemia") OR TI("iron deficiency anemia") OR AB("iron homeostasis") OR TI("iron homeostasis") OR AB("iron status") OR TI("iron status") OR AB("iron deficient non anemia") OR TI("iron deficient non anemia") OR AB("ferritin") OR TI("ferritin") OR AB("hepcidin") OR TI("hepcidin") OR AB("hemoglobin") OR TI("hemoglobin") OR AB("anemi*") OR TI("anemi*"))

AND ((MH "athletes, professional+") OR (MH "athletes, college+") OR (MH "female athlete triad+") OR (MH "athletes,female+") OR (MH "athletic training program+") OR (MH "athletes+") OR AB("athlete") OR TI("athlete") OR AB("athletes") OR TI("athletes") OR AB("collegiate") OR TI("collegiate") OR AB("recreational") OR TI("recreational") OR AB("endurance") OR TI("endurance") OR AB("physical performance") OR TI("physical performance") OR AB("military") OR TI("military") OR AB("soldier") OR TI("soldier") OR AB("solidiers") OR TI("solidiers"))

AND ((MH "dietary supplement+") OR (MH "dietary carbohydrate+") OR (MH "dietary fiber+") OR (MH "dietary supplementation+") OR (MH "probiotics+") OR (MH "synbiotics+") OR (MH "prebiotics+") OR (MH "dietary intervention+") OR AB("dietary iron") OR TI("dietary iron") OR AB("iron supplement") OR TI("iron supplement") OR AB("probiotic") OR TI("probiotic") OR AB("prebiotic") OR TI("prebiotic") OR AB("synbiotic") OR TI("synbiotic") OR AB("Lactobacillus plantarum 299v") OR TI("Lactobacillus plantarum 299v") OR AB("heme") OR TI("heme") OR AB("non-heme") OR TI("non-heme") OR AB("carbohydrate") OR AB("carbohydrates") OR TI("carbohydrate") OR TI("carbohydrates") OR AB("fiber") OR TI("fiber") OR AB("vitamin c") OR TI("vitamin c") OR AB("iron-rich food") OR TI("iron-rich food") OR AB("iron-fortified") OR TI("iron-fortified") OR AB("anti-inflammatory") OR TI("anti-inflammatory") OR AB("energy availability") OR TI("energy availability") OR AB("dietary intervention*") OR TI("dietary intervention*") OR AB("dietary supplement*") OR TI("dietary supplement*"))

<u>Embase Search Terms:</u>
('iron deficiency '/exp OR 'iron deficienc*':ti,ab OR 'iron deficiency anemia':ti,ab OR 'iron homeostasis':ti,ab OR 'iron status':ti,ab OR 'iron deficient non anemia':ti,ab OR 'ferritin':ti,ab OR 'hepcidin':ti,ab OR 'hemoglobin':ti,ab OR 'anemi*':ti,ab)
AND
('athlete '/exp OR 'physical performance '/exp OR 'student athlete'/exp OR 'athlete':ti,ab OR 'athletes':ti,ab OR 'collegiate':ti,ab OR 'recreational':ti,ab OR 'endurance':ti,ab OR 'physical performance':ti,ab OR 'military':ti,ab OR 'soldier':ti,ab OR 'soldiers':ti,ab)
AND
('diet therapy '/exp OR 'iron therapy '/exp OR 'probiotic agent'/exp OR 'carbohydrate '/exp OR 'dietary iron':ti,ab OR 'supplemental iron':ti,ab OR 'probiotic':ti,ab OR 'probiotics':ti,ab OR 'prebiotic':ti,ab OR 'prebiotics':ti,ab OR 'synbiotic':ti,ab OR 'synbiotics':ti,ab OR 'heme':ti,ab OR 'non-heme':ti,ab OR 'carbohydrate':ti,ab OR 'carbohydrates':ti,ab OR 'vitamin c':ti,ab OR 'iron-rich food':ti,ab OR 'anti-inflammatory':ti,ab OR 'energy availability':ti,ab OR 'fiber':ti,ab OR 'dietary intervention*':ti,ab OR 'dietary supplement*':ti,ab)
